# Two-dimensional Cu-based materials for electrocatalytic carbon dioxide reduction

**DOI:** 10.1016/j.isci.2024.109313

**Published:** 2024-02-23

**Authors:** Mingliang Hu, Li Li, Junjun Li, Kiran Zahra, Zhicheng Zhang

**Affiliations:** 1 Key Laboratory of Organic Integrated Circuit, Ministry of Education & Tianjin Key Laboratory of Molecular Optoelectronic Sciences, Department of Chemistry, School of Science, Tianjin University, Tianjin 300072, China; 2 Collaborative Innovation Center of Chemical Science and Engineering (Tianjin), Tianjin 300072, China

**Keywords:** Energy materials, Materials chemistry

## Abstract

Electrocatalytic CO_2_ reduction reaction (CO_2_RR) has emerged as a focal point in sustainable energy research, offering the potential for closed carbon cycle. Among numerous catalysts designed for CO_2_RR, two-dimensional (2D) Cu-based catalysts stand out for their remarkable performance in efficiently converting CO_2_ into high-value-added C_1_ and C_2+_ chemicals. Herein, we discuss the recent progress and challenges in the realm of CO_2_RR utilizing 2D Cu-based catalysts. The first section introduces various synthetic strategies, emphasizing the features and advantages of different techniques and proposing solutions to existing challenges. The second part outlines the reaction mechanism underlying the production of C_1_ and C_2+_ products on Cu-based catalysts, then summarizes applications of different types 2D Cu-based catalysts in CO_2_RR. Additionally, we evaluate the limitations of 2D Cu-based catalysts and propose improved strategies. Through this exploration of research advances and challenges, we hope to illuminate the path toward developing excellent CO_2_ electrocatalysts.

## Graphical abstract

**Figure:**
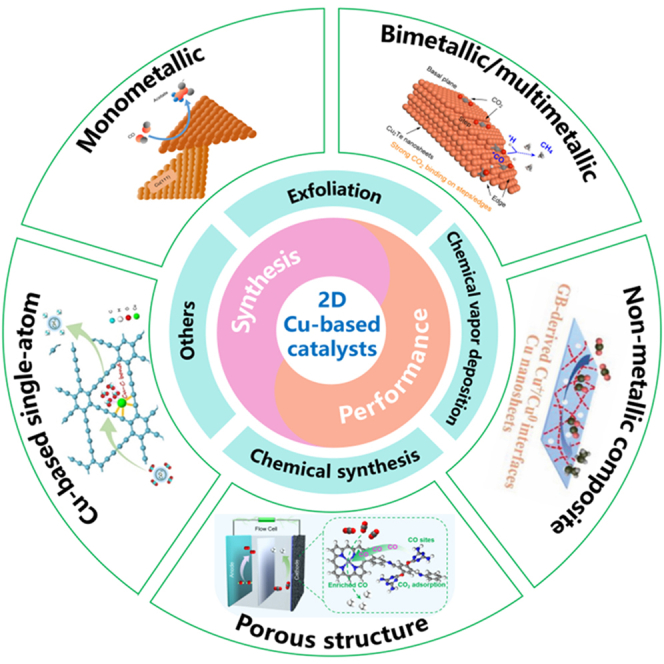

## Introduction

The effective utilization of energy is the basis for the sustainable development of human society. From harnessing fire to the recent reliance on fossil energy, power source innovations have fundamentally advanced science and technology. However, the unrestricted extraction and utilization of fossil energy, coupled with the direct emission of large amounts of CO_2_ into the atmosphere, have caused severe ecological damage. By July 2023, monitoring had indicated that atmospheric CO_2_ concentrations had reached 422 ppm, an increase of more than 50% from the pre-industrial revolution level of 280 ppm. The greenhouse effect, glacial melting, and ocean acidification caused by such high levels of CO_2_ in Earth’s atmosphere pose profound threats to the survival of all Earth’s species in the future.^1^^,^^2^^,^^3^^,^^4^^,^^5^ Researchers are working to close the carbon cycle loop to respond to this urgent situation by chemically converting CO_2_ into a recyclable and sustainable energy source via thermocatalytic, photocatalytic, and electrocatalytic processes.^3^^,^^4^^,^^5^ Due to the high thermodynamic stability of CO_2_, characterized by an activation energy of 750 kJ/mol for C=O bonding, conventional thermocatalytic processes need high-temperature conditions.^6^^,^^7^ For example, Cafer et al. reported that efficient CH_4_-CO_2_ reforming to syngas could be achieved under 800°C over Mo-doped Ni nanocatalyst at the edges of single-crystalline MgO.^7^ Besides, the energy consumption associated with high temperatures makes thermocatalytic process difficult to achieve carbon-negative utilization. The insecurity of the high-temperature environment and the rigorous restrictions on the catalyst are inevitable. CO_2_ reduction through photochemistry, mimicking natural photosynthesis, constitutes a green and sustainable approach known as the artificial photosynthesis process. Wang et al. prepared a g-C_3_N_4_/UiO-66 (Zr/Ce) photocatalyst that successfully converted CO_2_ into alcohol products (methanol and ethanol) under visible light irradiation. However, the yields of methanol and ethanol were only 54.71 μmol h^−1^ g^−1^ and 38.10 μmol h^−1^ g^−1^, respectively. What’s more, the light absorbance range of g-C_3_N_4_/UiO-66 (Zr/Ce) is concentrated below 450 nm, making it difficult to achieve the full solar spectrum utilization.^4^ The low optical quantum efficiency, product yield and selectivity make photocatalytic CO_2_ reduction difficult to realize on a large scale application in the short term.^8^^,^^9^^,^^10^ In order to avoid the dilemmas faced in thermocatalytic or photocatalytic process, CO_2_RR is a very promising option, which could be achieved under mild conditions and is not limited by geographic location or light conditions. CO_2_RR technology can convert CO_2_ into high-value-added chemicals using clean and renewable electricity, achieving zero CO_2_ emissions and closing the carbon cycle, displaying promising prospects for large scale application. To realize industrial-scale CO_2_RR, electrolytic cells, ion exchange membranes, gas diffusion electrodes, and catalytic materials need to be fully developed, among which the development of high-performance catalytic materials is crucial.^11^^,^^12^^,^^13^^,^^14^^,^^15^

In recent years, there has been a growing number of reports concerning various types of catalytic materials for CO_2_RR. Metals such as Ni, Ag, Zn, Au, and In can only produce C_1_ products like CO, CH_4_, HCOOH, and CH_3_OH in the CO_2_RR. Converting CO_2_ to carbon-based products fulfills a renewable energy process. However, typical gaseous C_1_ products (CO, CH_4_) are difficult to store and transport. Cu-based catalysts have the capacity to generate multi-carbon products such as C_2_H_4_, C_2_H_6_, CH_3_CH_2_OH and C_3_H_6_, demonstrating enormous potential for applications.^16^^,^^17^ C_2+_ products, especially the liquid phase (ethanol, acetate, propanol) have higher bulk energy density, and the process of storing and transporting is more convenient and safer. The generation of C_2+_ products over Cu-based catalysts could be attributed to the unique C-C coupling ability, i.e., direct dimerization of ∗CO and polymerization of ∗CO with protonated ∗CO (∗CHO or ∗COH).^18^^,^^19^^,^^20^ Until now, researchers have reported many instances of Cu-based catalysts in CO_2_RR for producing C_2+_ products. Various approaches have been employed to control the selectivity of product and improve the efficiency of the CO_2_RR, such as regulating the crystal facets, coordination structures, element doping, interface effects, and electrolyte pH value of Cu-based catalysts.^21^^,^^22^^,^^23^ However, Cu-based catalysts exhibit high overpotentials and slow reaction kinetics in the CO_2_RR, resulting in low Faradaic efficiency (FE). The FE of C_2_ products is difficult to reach the industrial production requirement of 70%, and the FE of C_3_ products is even lower than 10%. Furthermore, while Cu-based catalysts can generate C_2+_ products, the selectivity toward a specific product remains low due to the complex multi-electron transfer processes. For example, C_2_H_4_ and C_2_H_5_OH can be generated on the Cu-based catalyst with a 12e^−^ transfer process. Different intermediates coupling leads the number of electron transfers cannot be fixed, and therefore, 6e^−^, 8e^−^, 12e^−^, and 18e^−^ transfers can occur at the same time.^24^^,^^25^ Moreover, Cu-based catalysts in the electrocatalytic process suffer from activity loss owing to *in situ* microstructural changes, which causes challenges for attaining both high stability and selectivity in the CO_2_RR.^26^^,^^27^^,^^28^

**Figure 1:**
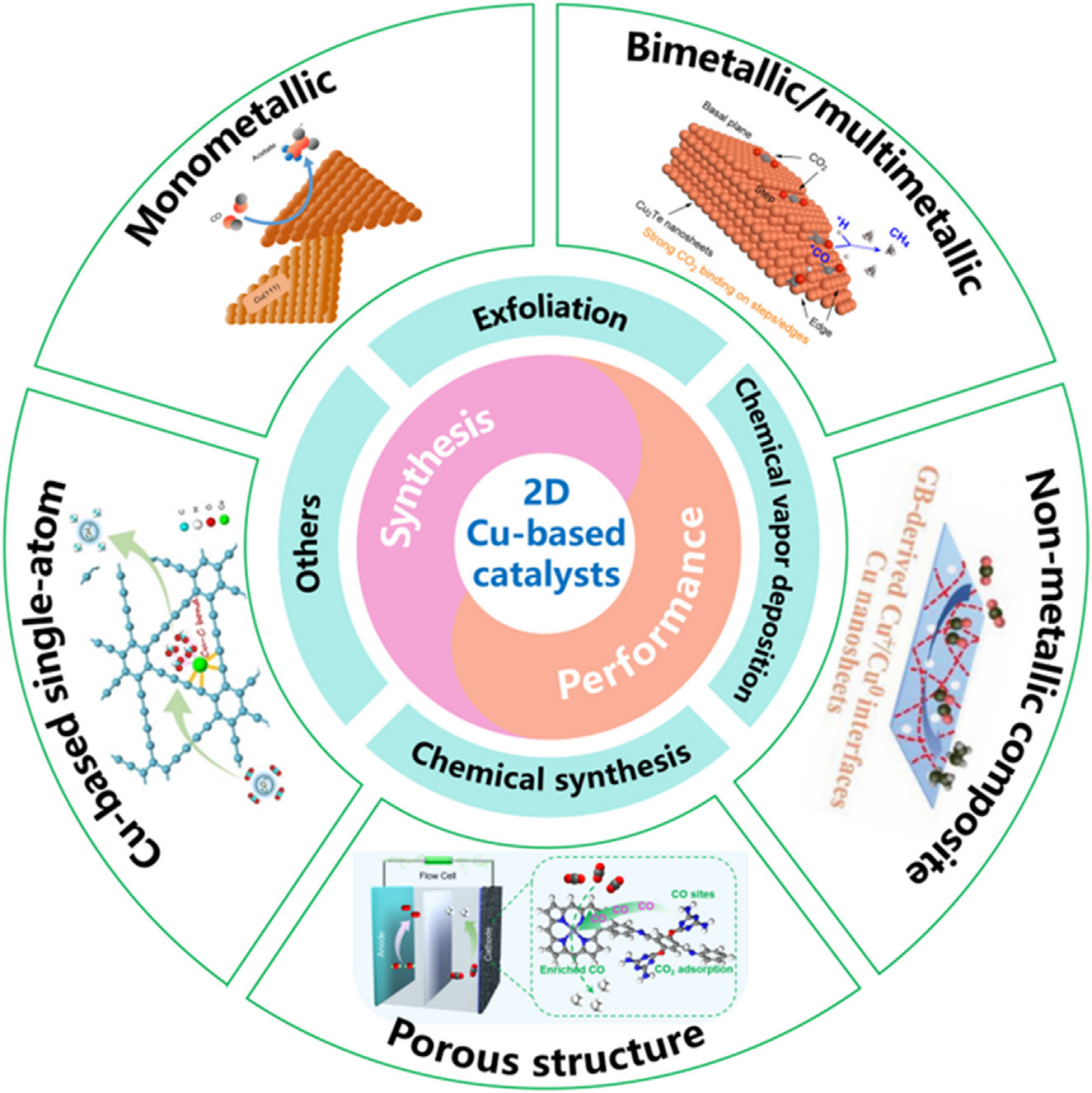
Overview of the main topics covered herein In this review, the recent advances, a variety of synthesis methods, and performance of 2D Cu-based catalysts for CO_2_RR are discussed. (Reproduced with permission from Ref.,^38^ © Nat. Catal. 2019; Reproduced with permission from Ref.; ^39^ © ACS Nano 2023; Reproduced with permission from Ref.; ^40^ © Adv. Sci. 2022; Reproduced with permission from Ref.; ^41^ © Angew. Chem. Int. Ed. 2021; Reproduced with permission from Ref.; ^42^ © Angew. Chem. Int. Ed. 2022).

An essential aspect of improving the efficiency of Cu-based catalysts for CO_2_RR is to increase the electron and proton transfer rates in the reaction system, which can effectively solve the problem of slow kinetics. Structural design is an effective strategy to enhance the electron and proton transfer rates of entire reaction system and to optimize the mass transfer process.^29^^,^^30^ 2D materials have been attracting worldwide attention since the development of graphene materials. The ultra-thin morphological appearance, large specific surface, and mechanical flexibility make 2D materials excellent prospects for electronic devices and electrocatalytic applications.^31^^,^^32^^,^^33^ Many studies have demonstrated that the 2D structure exposes abundant catalytic active sites, and the atomic-level thickness can effectively promote rapid electron transfer and reduce the diffusion resistance of mass transfer. In addition, the 2D structure ensures the homogeneity of reactant concentration and local microenvironment throughout the catalyst layer. Homogeneous local microenvironment can enhance the selectivity of products.^34^^,^^35^^,^^36^^,^^37^ In this paper, we summarize the design and preparation of Cu-based 2D materials and the application for CO_2_ electroreduction, which is expected to provide a reference for more efficient CO_2_RR catalysts (Figure 1). We first present various synthetic methods applied with the preparation of Cu-based 2D materials, including exfoliation, chemical vapor deposition (CVD), hydrothermal/solvothermal synthesis, template synthesis, interface synthesis and some unconventional strategies. Subsequently, the CO_2_RR performance of different types of Cu-based 2D materials are discussed in depth, including monometallic Cu nanosheets, bimetallic/multimetallic Cu-based catalysts, non-metallic composite Cu-based catalysts, porous Cu-based catalysts, and Cu-based single-atom catalysts. Finally, the current challenges in the preparation and application of Cu-based 2D materials are summarized, providing insights into realizing their large-scale applications.

## Strategy for the synthesis of Cu-BASED 2D materials

2D materials are sheet-like nanostructures that can extend infinitely in their theoretical lateral dimension while reaching a longitudinal of even atomic-level thickness. This huge aspect ratio leads to unconventional properties relative to 0-dimensional, 1-dimensional, or bulk materials, including physics, chemistry, electronics, optics, magnetism, etc. Since the successful development of graphene by Novoselov et al. in 2004,^43^ research on 2D materials has been a frontier of materials science research. New 2D materials have been successfully developed and applied in various fields, such as electronic and optical devices, catalytic materials, sensors, solar cells, biomedicine, environmentally friendly materials, energy storage, etc. At the present stage, there are more and more types of 2D materials, typically including graphene, metal nanosheets (NSs), metal sulfides, layered metal oxides, carbon and nitrogen compounds, layered double hydroxides (LDH), metal-organic frameworks (MOFs), covalent-organic frameworks (COFs), MXenes, black phosphorus (BP), organic polymers, organic-inorganic hybrid chalcogenides, and so on.^44^^,^^45^^,^^46^ Based on so many kinds of 2D structures, the design of Cu-based materials for CO_2_RR is beneficial to reducing the reaction overpotential, increasing the FE and obtaining more C_1_/C_2+_ products. A simple and feasible synthesis strategy of Cu-based 2D materials with stable products is the key to achieve this goal. In the past 20 years, the technology of 2D material preparation has expanded rapidly. A variety of strategies have been practiced and applied to achieve the controlled synthesis of 2D materials in terms of structure and size, including exfoliation, CVD, chemical synthesis, and so on.^31^^,^^47^ In this section, we highlight the different synthetic strategies in recent years for 2D Cu-based materials.

### Exfoliation strategy

**Figure 2:**
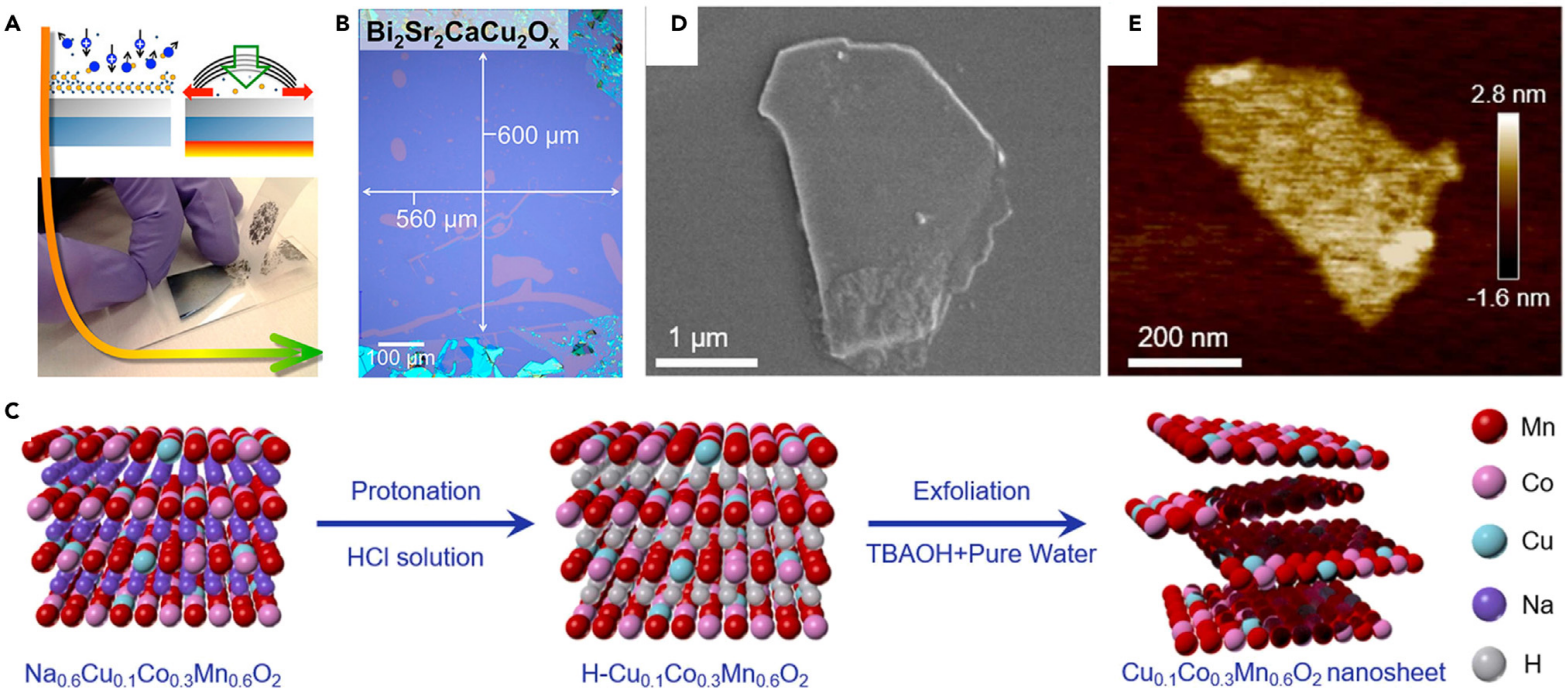
Exfoliation strategy for the synthesis of 2D Cu-based materials (A and B) (A) Mechanism diagram and operation process of exfoliation process. (B) Optical images of Bi_2_Sr_2_CaCu_2_O_x_ flakes prepared by exfoliation method.^48^(Reproduced with permission from Ref.,^48^ © ACS Nano 2015). (C‒E) (C) Schematic illustration of the preparation procedure of Cu_0.1_Co_0.3_Mn_0.6_O_2_ NSs. (D) SEM image of Cu_0.1_Co_0.3_Mn_0.6_O_2_ NSs. (E) AFM image of Cu_0.1_Co_0.3_Mn_0.6_O_2_ NSs.^49^(Reproduced with permission from Ref.,^49^ © Appl. Catal. B: Environmental 2023).

The monolayer graphene obtained via a mechanical exfoliation method used a tape adhesion process to get a monolayer structure from the surface of bulk graphite.^43^ With the further development of this method, the researchers accomplished the development of 2D materials such as hexagonal boron nitride(h-BN), molybdenum disulfide (MoS_2_), niobium diselenide (NbSe_2_), and black phosphorus (BP) etc. Exfoliation method is a typical top-down strategy for the preparation of 2D materials. The process is mainly based on the characteristics of the lamellar structure of bulk materials. Bulk precursors have strong intra-plane forces and weak inter-plane forces, facilitating 2D nanostructure formation via tape adhesion or liquid-phase ultrasonication. Exfoliation process does not rely on chemical substances or reactions, just on physical force processes to prepare 2D structures, thus preserving their original structure and intrinsic properties. The strong intra-interfacial covalent bonds or coordination structure and the weak van der Waals forces between the layers allow the exfoliated products to achieve ultra-high aspect ratios and atomic-level thicknesses. The 2D Cu-based materials preparation process has been successfully improved by applying the exfoliation method with the help of some auxiliary means. As shown in Figures 2A and 2B, Huang et al.^48^ achieved successful exfoliation of large-area and high-quality 2D Bi_2_Sr_2_CaCu_2_O_x_. By performing oxygen plasma cleaning of the substrate before exfoliation, environmental adsorbed species could be removed effectively. The accompanying thermal treatment process maximizes the uniform contact area at the interface between the crystal and the substrate. Li et al.^49^ successfully prepared Cu_0.1_Co_0.3_Mn_0.6_O_2_ NSs by protonation and exfoliation route (Figures 2C–2E), which included first synthesizing Na_0.6_Cu_0.1_Co_0.3_Mn_0.6_O_2_ with layered structure by a wet chemical process. Na_0.6_Cu_0.1_Co_0.3_Mn_0.6_O_2_ was used as the precursor for protonation and exfoliation, which achieves the transformation of Na^+^ to H^+^ through a deep proton exchange process with hydrochloric acid. H-Cu_0.1_Co_0.3_Mn_0.6_O_2_ obtained from the protonation process completes the liquid phase exfoliation process in tetrabutylammonium hydroxide solution with the help of shear force generated by mechanical stirring to finally obtain 2D Cu_0.1_Co_0.3_Mn_0.6_O_2_ NSs. To complete the transition of Cu-MOF from bulk to 2D structure, Qiao’s group^50^ proposed a method to drive the phase transition. Molecular shear under mild conditions to prepare MOF NSs with 2D structures by combining ultrasound assistance. A combination of solvent heat and solvent exchange prepared Cu-BDC as a precursor, then Cu_2_BDC was produced by shearing Cu-BDC precursor with ascorbic acid and ultrasound. The micron-sized flakes were transformed into nanoribbons with a width of 0.2–2 μm and thickness was 60 nm.

Similarly, increasingly assisted tools have been applied to the exfoliation process of 2D materials preparation. For example, mechanically assisted liquid phase exfoliation or sonic assisted liquid phase exfoliation processes use shear or sonic mechanical forces to break the van der Waals forces between layers to complete the exfoliation process of bulk materials. Ion insertion-assisted liquid phase exfoliation process involves the introduction of cations with a small radius between the layers of the bulk material to form an intercalation compound, thereby reducing the van der Waals forces between the layers and ultimately achieving the exfoliation process. The oxidation-assisted exfoliation process involves the oxidation of the bulk material by an oxidizing agent, and the oxidation process generates many surface functional groups which facilitate the completion of the exfoliation process. However, these exfoliation techniques still have many unsolved problems, such as low efficiency and uncontrollable products.^31^^,^^51^^,^^52^

### Chemical vapor deposition (CVD) strategy

The exfoliation strategy is a “top-down” synthesis route that requires the bulk material to have a laminar structure and therefore has obvious limitations in its application scenarios. However, the “bottom-up” synthesis strategy is based on the chemical reaction of precursors under specific conditions for the preparation of 2D structures and therefore has an extensive range of applications. CVD is a traditional “bottom-up” synthesis strategy. A typical CVD process begins with the presence of substrate material in a high temperature furnace and the circulation of a vapor phase precursor. The vapor phase precursor reacts and grows on the surface of the substrate under high temperature conditions to produce the desired 2D material. Compared to the exfoliation process, the controllability of the CVD process is significantly improved. The size, thickness, number of layers, and structural composition of the products can be regulated by controlling the growth temperature, gas flow rate, precursor composition, reaction pressure, or other parameters, and the purity and yield are improved accordingly.^51^^,^^52^^,^^53^ Typically, as reactants for the CVD process, precursors affect the composition of the 2D material directly. Temperature setting determines whether the chemical reaction between the precursors can take place. In addition, different growth rates of lateral and vertical sizes were observed at different reaction temperatures. The gas flow rate will guide the mass transfer process, affecting the final morphology. Other parameters, such as atmosphere and carrier gas pressure, affect the nucleation process and final morphological structure. Appropriate parameter settings are crucial to a successful CVD process.^54^

**Figure 3:**
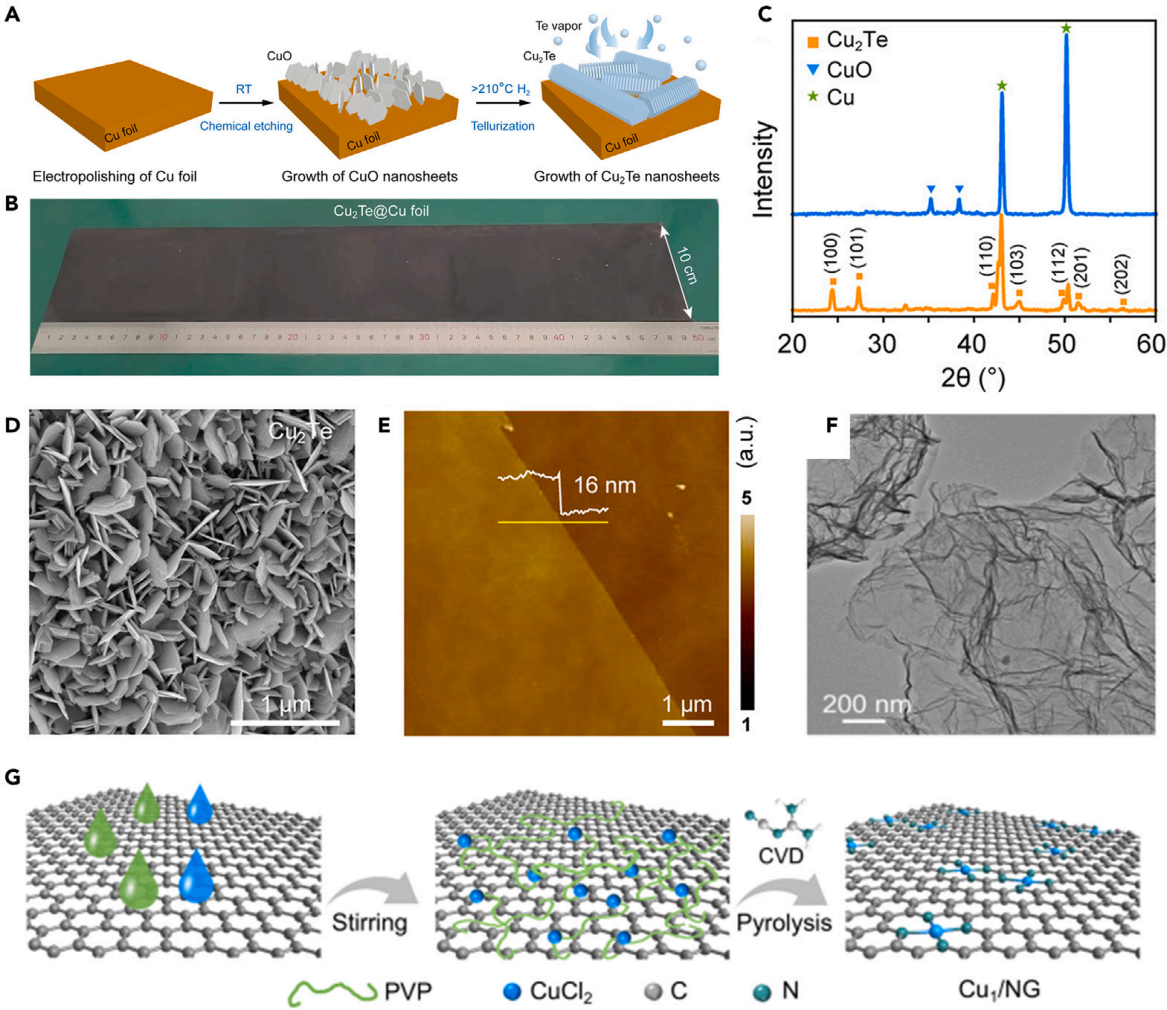
CVD strategy for the synthesis of 2D Cu-based materials (A‒E) (A) Schematic illustration for CVD growth of 2D Cu_2_Te NSs. (B) Photograph of a 10 × 50 cm^2^ Cu_2_Te@Cu foil. (C) XRD spectra of 2D Cu_2_Te NSs. (D) SEM images of 2D single-crystal Cu_2_Te NSs. (E) AFM image of 2D single-crystal Cu_2_Te NSs with 50 nm edge thickness of Cu_2_Te NSs.^39^(Reproduced with permission from Ref.,^39^ © ACS Nano 2023). (F and G) (F) TEM image of Cu_1_/NG. (G) Synthesis process for the Cu_1_/NG catalyst.^55^(Reproduced with permission from Ref.,^55^ © Angew. Chem. Int. Ed. 2022).

Since the CVD process was first successfully used for the fabrication of single-layer graphene, this method has evolved and gradually become an established strategy for the fabrication of 2D nanomaterials. The synthesis of graphene, hexagonal boron nitride and 2D metal sulfides has been successfully achieved using the CVD process. It has been reported that 2D Cu-based materials can be prepared using the CVD process. As shown in Figures 3F and 3G, Li’s group^55^ prepared a nitrogen-doped graphene-loaded Cu catalyst (Cu_1_/GN) with a 2D structure by CVD method. Specifically, a mixed powder of CuCl_2_, polyvinylpyrrolidone (PVP) and graphene as a substrate material was first prepared, and then the dicyandiamide and the prepared mixed powder were placed in two porcelain boats in a high-temperature furnace. The NH_3-x_ vapor generated from the *in situ* decomposition of the dicyandiamide diffused with the nitrogen flow and reacted with the CuCl_2_/PVP/graphene mixture to finally obtain Cu_1_/NG. The loading of metallic Cu could be regulated by the amount of precursor metal salts to eventually obtain a series of catalysts with different site densities. Recently, Chen’s group^39^ reported the controlled growth of large-area 2D single-crystal hexagonal phase Cu_2_Te NSs on the surface of a commercial copper foil as substrate material by electrochemical etching and CVD processes (Figures 3A–3E). The growth process of Cu(OH)_2_ nanowires to 2D CuO NSs was completed by electrochemical etching in an alkaline solution. Followed by vapor deposition of Te using CuO as a self-sacrificing template to finally obtain 2D single-crystal Cu_2_Te NSs array structures. Combining the two steps of electrochemical etching and CVD can precisely regulate the size and thickness of 2D layered Cu_2_Te. In the CVD process, the high melting point of the Cu metal source makes it difficult to vaporize. So, the deposition process and obtaining a perfect 2D structure become difficult, resulting in an inhomogeneous crystalline shape. Yang et al.^56^ developed a salt-assisted CVD strategy to cope with the dilemma of CuO with a high melting point and low saturation vapor pressure. NaCl, which has a melting point more compatible with CuO, was chosen to form halide oxides with CuO and thus raise the saturation vapor pressure. Moreover, NaCl also acts as a passivating agent to inhibit (001) crystal growth in the crystalline direction, which is beneficial for the fabrication of a high-quality single-crystal Cu_9_S_5_ NS. The CVD process has enormous potential for industrial-scale electronic or optoelectronic device preparation due to its superb controllability in terms of preparation, yet its high cost is still a barrier that needs to be overcome.

### Chemical synthesis strategy

Similar to the CVD process, the fabrication of 2D nanomaterials by chemical synthesis can also be implemented by a “bottom-up” strategy. The chemical synthesis process has excellent product tunability and enables the synthesis of ultrathin 2D materials with limited thickness, offering potential for industrial-scale applications. Compared to exfoliation and CVD processes, chemical synthesis is a more flexible technique for preparing a variety of novel 2D materials. To date, there are no universal fundamentals for chemical synthesis methods. Chemical synthesis strategies can only be classified according to their characteristics, such as hydrothermal/solvothermal synthesis, template method, interface/surface synthesis, electrochemical deposition, self-assembly method, and co-precipitation, etc. These methods can be used to flexibly fabricate various Cu-based 2D materials.^31^^,^^51^^,^^52^^,^^53^ This section will focus on several different chemical synthesis processes and their applications in the synthesis of Cu-based 2D materials.

### Hydrothermal/solvothermal synthesis strategy

**Figure 4:**
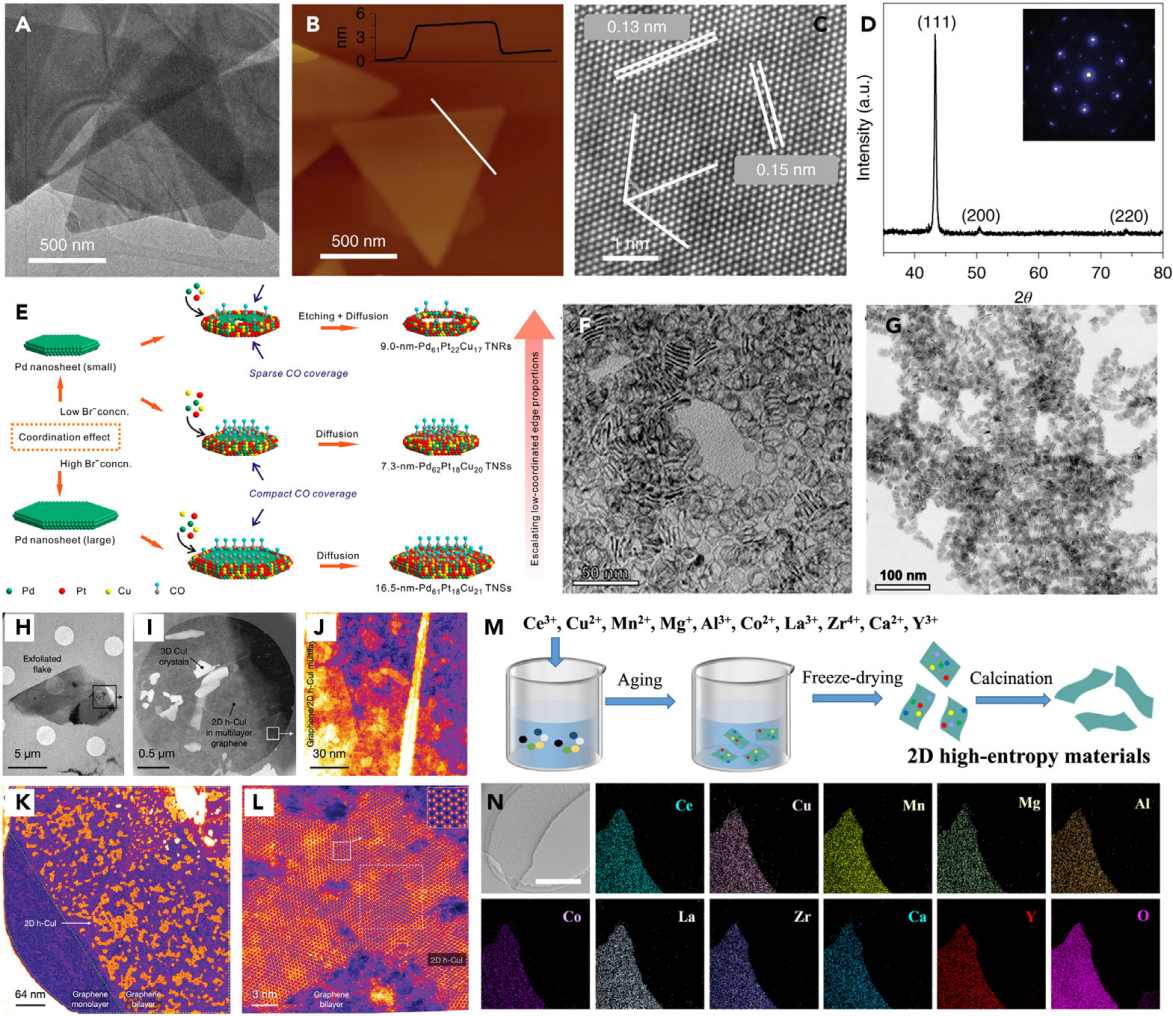
Hydrothermal/solvothermal and template strategies for the synthesis of 2D Cu-based materials (A‒D) (A) TEM image of triangular Cu NSs. (B) AFM image of Cu NSs. (C) HRTEM image of a Cu NSs. (D) XRD pattern of Cu NSs assembled on a Si wafer (Inset: SAED pattern of Cu NSs).^38^(Reproduced with permission from Ref.,^38^ © Nat.Catal. 2019). (E‒G) (E) Schematic illustration for ultrathin 2D PdPtCu nanostructures. (F) TEM image of Pd_61_Pt_22_Cu_17_ TNRs. (G) TEM image of Pd_62_Pt_18_Cu_20_ TNSs.^59^(Reproduced with permission from Ref.,^59^ © Nano Lett. 2020). (H‒L) (H) Bright-field TEM image of a single graphene/h-CuI flake suspended on a TEM support film. (I-J) HAADF images of the flake edge. The orange and yellow contrast values in (J) are h-CuI. (K) HAADF overview image of monolayer h-CuI crystals encapsulated in a bilayer graphene sandwich. (L) Atomically resolved HAADF closeup of a single 2D h-CuI crystal with a magnifying inset in the top right corner.^60^(Reproduced with permission from Ref.,^60^ © Adv. Mater. 2022). (M and N) (M) The preparation diagram of 2D high-entropy materials. (N) TEM image and elemental mapping images of 2D Ce_1_Cu_1_Mn_1_Mg_1_Al_1_Co_1_La_1_Zr_1_Ca_1_Y_1_O_x_.^3^(Reproduced with permission from Ref.,^3^ © Nat. Commun. 2023).

Hydrothermal or solvothermal processes are simple and usually carried out in sealed Teflon reactors. The hydrothermal and solvothermal methods use water and organic solvents respectively as reaction medium. Compared with other synthesis methods, the reaction temperature of hydrothermal or solvothermal processes can surpass the solvent’s boiling point, which raises the pressure of the closed reaction system and improves the crystallinity of the nanocrystals. At the same time, the solubility and reactivity of the reactants increased, and the physicochemical properties of the whole reaction system also changed radically. The nucleation and growth steps can be modulated by adjusting parameters such as the ratio of metal precursors, type of ligand or surfactant, reaction temperature and time during hydrothermal/solvothermal process, thus achieving adjustable morphology and size. For example, surface surfactants can significantly reduce the energy of a desired facet, making the crystal more stable. Besides, the crystals can grow faster in certain directions with specific surfactants, resulting in different shapes and sizes. Surfactants can also modulate the size of nanomaterials by controlling nanoparticle nucleation, growth rate, and aggregate size.^57^ Given these merits, hydrothermal/solvothermal procedures have been widely exploited for the preparation process of 2D materials. In 2016, Gao et al.^58^ showed a Co-based metal nanosphere with only four atomic thicknesses by a solvothermal process. Co-based nanosphere effectively facilitated the preparation of formic acid by CO_2_RR via modulating the partial Co oxidation on the surface. Co(acac)_3_ was used as a metal precursor, and a mixture of dimethylformamide, H_2_O, and n-butylamine as the solvent, a four-atom-thick Co metal layer with a partially oxidized state was successfully synthesized by reacting in a sealed Teflon reactor at 220°C for 3 h. When the reaction time extended to 48 h, the oxidized state of Co disappeared, but the four-atom-thick morphology was still retained, and the metallic Co thin layer was obtained. Hydrothermal/solvothermal processes are also well suited for Cu-based 2D materials. For example, Wang et al.^37^ prepared a 2D flake CuO catalyst by a simple hydrothermal tool that achieved CO_2_ electrocatalytic reduction to C_2+_ products at industrial current densities with neutral pH. The catalyst preparation used pure water as the solvent, and the copper sulfate precursor was dissolved and precipitated by potassium hydroxide and ammonium hydroxide. Then the reaction was carried out at 80°C for 12 h in a sealed vessel, and the resulting product was thermally decomposed to give the desired flake CuO. This synthetic approach without any surfactant simplifies the synthesis and costs less. However, it is hard to achieve effective modulation for products requiring a specific morphology. Adding surfactants during hydrothermal/solvothermal processes can lead to particular morphologies or structures. And specific exposure of crystalline facets or morphologies is often critical for product selectivity. For example, in 2019, Kang’s group^38^ demonstrated a high acetate selectivity and activity in CO electroreduction by designing a triangular-shaped 2D Cu NSs that exposed specific {111} crystallographic facets (Figures 4A–4D). Deionized water, Cu(NO_3_)_2_·3H_2_O, l-ascorbic acid, cetyltrimethylammonium bromide and hexamethylenetramine were used as the solvent, metal precursor, reducing agent, and surfactants, respectively. The reaction carried out at 80°C for 3 h in a sealed reactor to obtain regular of triangular Cu NSs with a thickness of only 5 nm and highly selective exposure of Cu {111} crystal planes. Compared with single-metal Cu-based catalysts, multi-metal Cu-based catalysts exhibit more functional and synergistic effects, which can also be obtained via the hydrothermal/solvothermal process. As shown in Figures 4E and Xie et al.^59^ reported an ultrathin 2D PdPtCu trimetallic NSs and nanorings prepared by a one-pot solvothermal way (Figures 4F and 4G). Precise size regulation and shape selection of PdPtCu NSs and nanorings were achieved through integrated control of reaction kinetics, surface modification and selective etching.

### Template synthesis strategy

In the field of materials synthesis, specifically inorganic nanomaterials, the template method is a very effective strategy for the synthesis of nanomaterials with specific morphological and structural demands. Researchers use previously prepared bulk structures, 2D structures or materials with a confined spatial structure as templates for the synthesis of various 2D materials. Typically, the templates possess specific active sites that can interact with metal precursors and related mesophases, or have a defined spatial structure that can accommodate the metal precursors and complete their directed growth process.^61^ Exemplarily, Zhao et al.^62^ used iron trichloride solution as a metal precursor, and successfully prepared 2D Fe NSs with only a single-atom thickness on a graphene template. Among them, the interaction between C atoms and Fe atoms at the edge of graphene pores plays an essential role in controlling the growth of Fe NSs. As shown in Figures 4H‒4L, Kimmo et al.^60^ synthesized stable 2D cuprous iodide in the interlayer space of graphene at room temperature, a 2D structure that occurs only in a layered form at high temperatures between 645 and 675 K. This process takes advantage of the widely spaced spatial structure of graphene oxide multilayers, which allows iodine and copper atoms to diffuse into the gaps and grow, and applies high pressure to the intercalated material, leading to its stabilization. The procedure of building materials based on specific 2D structures such as graphene, graphene oxide, reduced graphene oxide, layered double hydroxide (LDH), transition metal sulfides, etc. is often referred to as the hard-template method. Hard-template are usually structurally stable and provide strong support during the synthesis step, but the removal of templates is always unsatisfactory. The soft-template method uses surfactants as templates for the assembly of 2D materials in solution, and its removal process is relatively simple. Recently, Ye et al.^3^ proposed a general and batch method for the preparation of high-entropy 2D oxides. Using polyvinylpyrrolidone (PVP) as a soft template to synthesize six-to eleven-membered high-entropy 2D materials (Figures 4M and 4N). Among these, the Cu-based high-entropy 2D materials maintain structural stability between 400°C and 800°C and have excellent CO_2_ hydrogenation properties. Firstly, PVP and metal precursors were dissolved into a mixed solution, followed by an aging process in which PVP self-assembles to form 2D micelles, and then undergoes rapid freeze-drying via liquid nitrogen to form 2D PVP micellar solids loaded with multiple metal ions. Finally, a series of high-entropy 2D materials were obtained with the removal of PVP templates by a high-temperature annealing process in air. Additionally, a unique synthetic process called the self-sacrificing template method can provide growth-limited space and participate in the growth process for 2D materials as precursors. Xie et al.^63^ synthesized chalcopyrite-type CuInSe_2_ NSs with a thickness of only 2.0 nm by the self-sacrificing template method. They used 2D CuSe as the template due to the well-matched lattice between CuSe and CuInSe_2_, which facilitates the cation exchange process of Se and In during the reaction. Finally, the (001) crystalline plane of CuSe NSs transformed into CuInSe_2_ ultrathin NSs with (112) crystalline plane.

### Interface synthesis strategy

**Figure 5:**
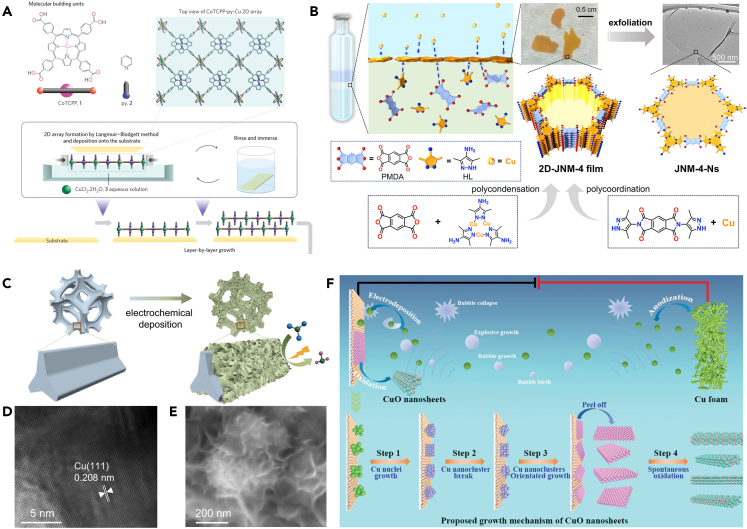
Interface and electrochemical strategies for the synthesis of 2D Cu-based materials (A) Schematic illustration of the fabrication method of CoTCPP-py-Cu.^65^(Reproduced with permission from Ref.,^65^ © Nat. Mater. 2010). (B) Schematic illustration of inter-facial synthesis involving three active monomers and two types of polymerization reactions.^66^(Reproduced with permission from Ref.,^66^ © J. Am. Chem. Soc. 2022). (C‒E) (C) Schematic diagram of CuCo alloy electrodeposition on nickel foam’s surface. (D) HRTEM image of Cu_50_Co_50_. (E) SEM image of Cu_50_Co_50_.^69^(Reproduced with permission from Ref.,^69^ © Nat. Commun. 2022). (F) Schematic illustration of ultrasonication-assisted electrodeposition of CuO NSs.^40^(Reproduced with permission from Ref.,^40^ © Adv. Sci. 2022).

Apart from the template synthesis strategy which can facilitate controlled growth of 2D materials within confined spaces, the interfaces (such as gas/liquid interface, liquid/liquid interface, gas/solid interface, liquid/solid interface) can also provide constrained environments that assist in guiding molecules or precursors for oriented growth. In the 1930s, Langmuir and Blodgett succeeded in obtaining 2D membrane materials by dispersing amphiphilic molecules on the water surface and arranging them into single molecular layers by gradual compression. This technique, known as the Langmuir-Blodgett (LB) method, is used for developing 2D materials based on the air-liquid interface. By this way, Thomas et al.^64^ synthesized the first monolayer coordination metal 2D structures at the water/air interface by confining the ligand at the interface to react with a metal salt dissolved in water, eventually leading to monolayer polymer NSs. In 2010, Makiura et al.^65^ reported a successful way of a 2D Cu-based MOF based on the LB process (Figure 5A). First, an aqueous CuCl_2_·2H_2_O solution was filled in a polytetrafluoroethylene Langmuir bath as the substrate phase. Then CoTCPP and pyridine dissolved in chloroform/methanol solvent were extended by a micro-conditioning syringe over the CuCl_2_·2H_2_O aqueous phase interface to generate 2D Cu-mediated CoTCPP arrays (CoTCPP-py-Cu). Finally, 2D CoTCPP arrays were stacked by deposition on Si(100) (or quartz) substrates in a continuous layer-by-layer growth procedure to obtain MOF nanofilms of the desired thickness. The gas/liquid interface synthesis strategies are usually performed under milder reaction conditions. MOF NSs synthesis by interfacial coordination polymerization. The preparation of 2D COF by interfacial organic condensation at room temperature. However, interfacial perturbations affect the formation of 2D structures under solvent heat conditions. Besides, the heating process also causes competition between different active components, matching the kinetics of different reactions and changes in the compatibility of coordination and organic condensation. So the synthesis of 2D multi-component MOF or COF by simple gas/liquid interfaces cannot be easily achieved. To realize an interfacial synthesis method of 2D NSs with multiple active ingredients and different types of polymerization reactions, as shown in Figure 5B, Li’s team^66^ successfully constructed defect-free, highly crystalline and large-area 2D MOF films by completing both ligand polymerization and polycondensation processes at the interface. Li et al. built a particular three-layer interfacial structure by setting a mixture of N,N-Dimethylformamide (DMF) and pyridine containing 3,5-dimethyl-4-amino-pyrazole and pyromellitic dianhydride (PMDA) as the bottom layer. DMF and ethanol solvent mixture set as the middle layer, and Cu(NO_3_)_2_·3H_2_O ethanol solution as the top layer. The three-layer interfacial structure finally yielded large-sized highly crystalline 2D imide linked MOF films by a solvothermal process, a self-supported 2D MOF NSs with high aspect ratio up to 2000:1 and ultrathin thickness of about 1.7 nm are obtained by easy exfoliation. Fabrication of interfaces by polymer coating of Cu-based structures is a strategy to enhance catalytic performance. Such interfaces can activate reactants and stabilize key intermediates. For example, Gewirth et al. used amino-containing organic polymers to modify Cu, and the modulation of the polymers with different degrees of alkylation revealed that -NH_2_ was the key to enhancing ethylene selectivity. -NH_2_ can promote CO_2_ adsorption, leading to an increase in local CO_3_^2−^ and enhancing the probability of C-C coupling.^67^ Zhuang et al. used 7,7,8,8-tetracyanoquinodimethane (C_12_H_4_N_4_) as a coating polymer. The interface can locally enrich and activate CO_2_, resulting in a selectivity of almost 50% for C_2_H_4_ and 80% for C_2_.^68^

### Electrochemical synthesis strategy

Beyond the previously mentioned exfoliation, CVD, and hydrothermal strategies, Cu-based 2D materials can also be accomplished by the electrochemical synthesis process,^40^^,^^69^^,^^70^ which is simple, controllable, fast, inexpensive, and can be performed at room temperature. Electrochemical synthesis generally adopts a two or three-electrode system. The oxidation and reduction process can be tuned by controlling the applied potential or deposition current to the working electrode. Parameter settings such as current mode (DC/pulse), overpotential, and electrolyte concentration can regulate the morphology and size of the final product. More importantly, working electrodes with special structures can carry out electrochemical synthesis. Nickel foam, copper foam, metal mesh, carbon cloth, and various nanorods, nanowires, and nanotubes can be used as substrate materials to obtain functionalized nanomaterials.^71^ The most prominent advantage is that electrochemical synthesis can directly complete the preparation of electrocatalytic integrated electrodes, eliminating the original electrode coating, which significantly reduces costs and operational difficulties. Traditional electrode preparation requires coated catalyst powder on the electrode surface with complex procedures and has the following problems: (1) easy to fall off; (2) high interfacial contact resistance; and (3) low active site utilization. The integrated electrode prepared directly on the conductive substrate by electrochemical synthesis can solve the above problems. The seamless contact can avoid the dropping of the catalyst and eliminate the interfacial resistance. With appropriate electrochemical synthesis parameters controlling or preparing them on the porous substrate, the integrated electrode can get a high specific surface area, increasing active site utilization significantly. Integrated electrodes can prepared at room temperature, which is favorable for scale-up production.^72^^,^^73^^,^^74^ Huang et al.^70^ synthesized 2D Cu/p-CuO/NiCo-P NSs by a three-electrode system. Cu mesh (400 mesh, 2 × 2 cm^2^), Pt foil, and Ag/AgCl were used as the working, counter, and reference electrodes, respectively. The Cu(OH)_2_ nanowire arrays were grown on the Cu mesh substrate in a 2 M KOH solution under a 15 mA cm^−1^ current density. Finally, porous CuO nanowire arrays (Cu/p-CuO NWAs) were obtained by further calcination. Subsequently, Cu/p-CuO NWAs were used as the working electrodes, keeping Pt foil and Ag/AgCl as the counter and reference electrodes, respectively. The electrolyte was mixture of Co(NO_3_)_2_·6H_2_O, Ni(NO_3_)_2_·6H_2_O, and NaH_2_PO_2_·H_2_O dissolved in ethanol and deionized water. Electrodeposition used cyclic voltammetry with a scan rate of 5 mV s^−1^ to obtain Cu/p-CuO/NiCo-P. The electrochemical deposition can also prepare 2D structures on many different substrate materials, such as carbon cloth, copper foil, nickel foam, FTO, etc. As shown in Figures 5C–5E, Sun’s group^69^ used electrocatalytic nitrate inert material nickel foam as the substrate to prepare 2D CuCo NSs by a two-electrode system. Nickel foam was chosen as a substrate because of its smooth surface and good conductivity. The electrolyte was an aqueous solution of precursors for the deposited metals, including trisodium citrate pentahydrate, CuSO_4_, and CoSO_4_. Adjusting the ratio of different metals in the precursors could regulate the composition and structure of products. The electrodeposition process was carried out at a current density of 50 mA cm^−2^ for 300 s to obtain 2D CuCo NSs. Several auxiliary approaches are exploited and applied to further enhance the properties of Cu-based 2D materials obtained by electrochemical synthesis. For instance, Zhang et al.^40^ developed an ultrasonic-assisted electrodeposition method to achieve large-scale synthesis of CuO NSs (Figure 5F). Similarly, using a two-electrode system, the whole electrodeposition bath is placed in a 160 W ultrasonic bath. Electrolyte solution adopts an alkaline KOH solution, when the electrochemical oxidation of copper foam at the anode occurs to generate Cu^2+^ ions directly will generate [Cu(OH)_4_]^2-^ complexes in the alkaline solution. [Cu(OH)_4_]^2-^ complexes are subsequently reduced at the cathode to obtain Cu NSs and exfoliated under ultrasonic action, and oxidized in KOH solution to become CuO NSs. Besides the ability to achieve the exfoliation process of Cu NSs by ultrasonication, the reaction time of Cu anodic oxidation can be regulated, and increasing the ultrasonic power can increase the yield of electrodeposited CuO NSs.

### Other synthesis strategies

**Figure 6:**
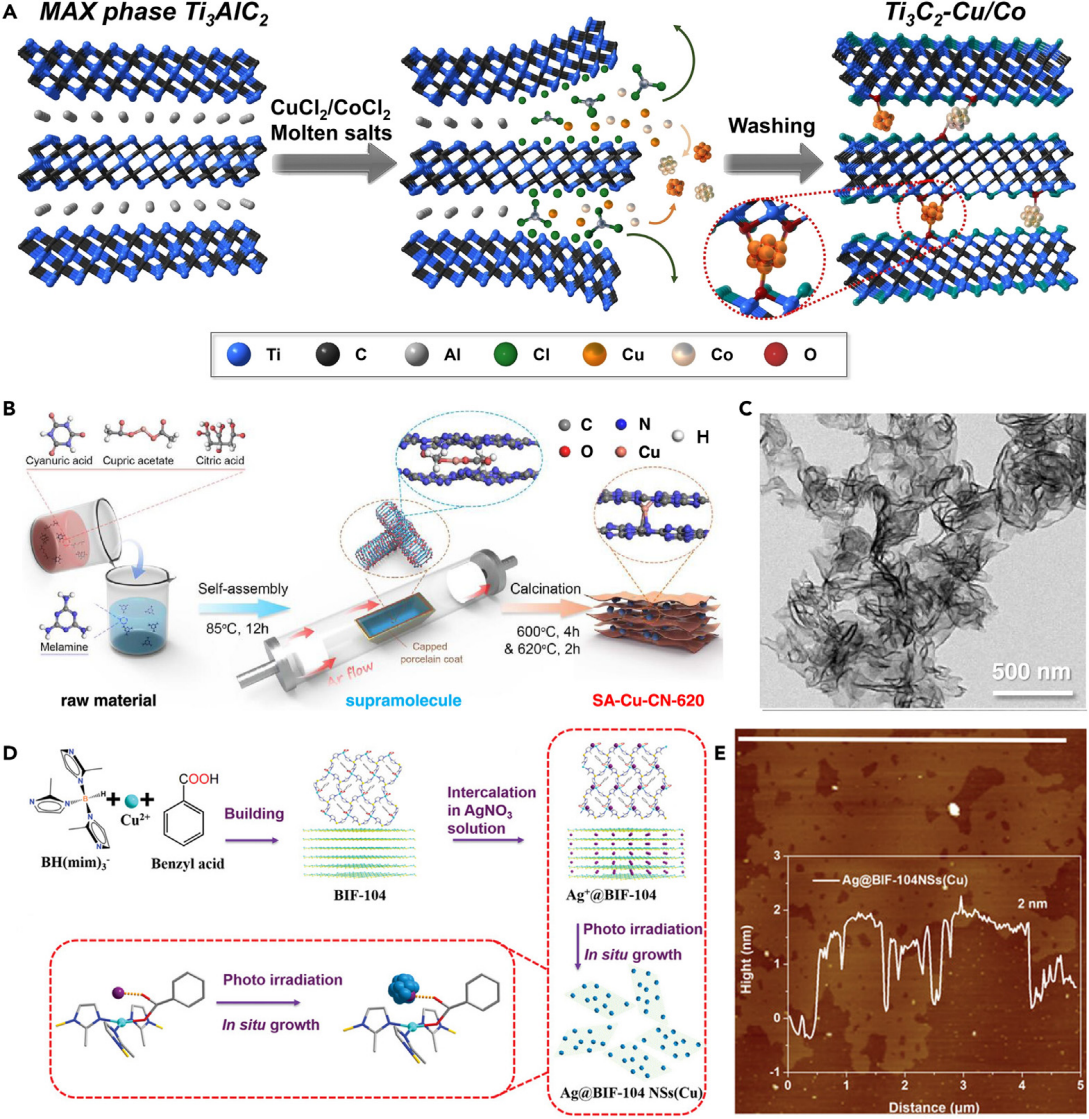
Unconventional strategies for the synthesis of 2D Cu-based materials (A) Preparation of Ti_3_C_2_-Cu/Co hybrids.^76^(Reproduced with permission from Ref.,^76^ © Angew. Chem. Int. Ed. 2021). (B and C) (B) Illustrated preparation of SA-Cu-CN-620. (C) TEM image of SA-Cu-CN-620.^79^(Reproduced with permission from Ref.,^79^ © ACS Catal. 2023). (D‒E) (D) Schematic illustration of the synthesis procedure of Ag@BIF-104 NSs(Cu). (E) AFM image and corresponding height of as-synthesized Ag@BIF-104 NSs(Cu).^80^(Reproduced with permission from Ref.,^80^ © Adv. Energy Mater. 2023).

In addition to the mentioned synthetic strategies for 2D materials, some unique synthetic processes have been developed and completed for the synthesis of 2D structures. For example, in 2022, Yang et al.^75^ successfully prepared 2D N-doped carbon NSs loaded with Fe single-atom catalysts by the molten salt process. The advantage of the molten salt process over the previously mentioned synthesis methods is the high synthetic efficiency. The liquid phase reaction environment presented by the molten state of the salt when the temperature exceeds the melting point (Tm) of the salt provides a suitable dissolved phase environment for substances with low solubility in water or other solvents. At the same time, salt mixing with different melting points can also change the melting point. A combination of different salts can regulate the reaction temperature and ultimately control the catalyst preparation process. Surplus salts during the reaction can be removed readily by water washing. As shown in Figure 6A, Pang et al.^76^ applied the molten salt method to prepare 2D Cu-based Mxene by adding copper salt (CuCl_2_-2H_2_O) to MAX phase Ti_3_AlC_2_, mixing and ground well before adding NaCl and KCl to continue milling and blending well, and finally obtaining 2D Cu-based Mxene by completing the molten salt process at a high temperature of 750°C. The nanocrystal self-assembly process has evolved as a stable way to generate internal nanostructures, e.g., Ma et al.^77^ prepared Cu/Au heterojunctions by electrostatic self-assembly of elaborate positively charged Cu nanoparticles and negatively charged Au nanoparticles, achieving an FE of 60% for electrocatalytic CO_2_ reduction to ethanol at a current density of more than 500 mA cm^−2^. Self-assembly through non-covalent interactions (e.g., van der Waals forces, electrostatic interactions, hydrogen bonding) between low-dimensional Cu nanocrystals (nanoparticles or nanowires) to accomplish the preparation of ultrathin Cu-based 2D nanostructures is a very promising strategy. In 2014, Yang’s group^78^ used 1-dodecanethiol ligand-coated Cu nanoclusters in colloidal solution for self-assembly. By modulating dipole gravitational forces between Cu nanocrystals and van der Waals forces between ligands to achieve the preparation of ultrathin Cu nanoribbons at the individual nanocrystal scale. Aside from the ability of the pre-formed nanocrystals to fulfill the self-assembly process, recently Liu et al.^79^ constructed a single-atom copper-bridged C_3_N_4_ (SA-Cu-CN-620) catalyst with abundant nitrogen vacancies based on the thermal condensation reaction between the copper precursor self-assembled supramolecule and melamine-cyanuric acid monomer (Figures 6B and 6C). The Cu precursor consists of citric acid and copper acetate, which can be introduced into the melamine-cyanuric acid layered structure through the hydrogen bonding self-assembly between citric acid and melamine-cyanuric acid. This method leads to 2D Cu-CN flakes by the thermal condensation process eventually. Some other simple coordination synthesis methods have also been employed to synthesize Cu-based 2D materials. Shao et al.^80^ formed a twisted tetragonal cone configuration by coordinating Cu^2+^ ions with N atoms on imidazole ligands and O atoms on benzoate salts to create a honeycomb network and 2D structure (Figures 6D and 6E). Li’s group^81^ added terephthalic acid as a ligand and triethylamine as a polymerization inhibitor to a mixed metal precursor solution of copper nitrate, nickel nitrate, and zirconium chloride in proportion. Under the ultrasonic effect, the metal ions coordinated with the carboxylic acid junction of terephthalic acid and grow directionally in two dimensions under the confinement of triethylamine, ultrathin 2D NiZrCu-BDC is acquired finally.

Advantages and shortcomings of the various synthesis methods are summarized in Table 1.

**Table 1:** Summary of different synthesis strategies

Strategy classification	Advantages	Shortcomings	Materials	Reference
Exfoliation	Straightforward	Low efficiency	graphene	Novoselov et al.^43^
CVD	Controllable	Harsh conditions	Cu_1_/GN	Wang et al.^55^
Hydrothermal/solvothermal	Universal	Poor durability	Flake CuO	Wang et al.^37^
Template	Controllable	Low efficiency	Fe NSs	Zhao et al.^62^
Interface	Controllable	Complicated	Cu-based MOF	Makiura et al.^65^
Electrochemical	Moderate condition	Complex equipment	CuCo NSs	Sun et al.^69^
Molten salt	High efficiency	Harsh conditions	Cu-based Mxene	Pang et al.^76^
Self-assembly	Low-cost	Hard control	Cu nanoribbons	Yang et al.^78^
Coordination	Simple	Poor stability	NiZrCu-BDC	Yang et al.^81^

## 2D Cu-BASED materials for CO_2_RR

The CO_2_RR has undoubtedly been an enthusiastic research area with a mild and controlled reaction environment. Researchers have fabricated a series of catalysts to convert electrical energy (available through a green renewable process) to chemical energy. Metal-based catalysts have shown excellent performance in the CO_2_RR process, with Zn, Au, Pd, and Ag as the representative metal-based catalysts successfully achieving the electroreduction of CO_2_ to CO, and on In, Bi, and Pb mainly generating formate salts.^18^^,^^19^^,^^20^ Cu-based catalysts are known for their ability to exhibit unique C-C coupling during the CO_2_RR, resulting in the formation of more valuable C_2+_ products. This enhances the economic viability of the CO_2_RR, making various Cu-based electrocatalytic catalysts a popular choice. The products of this process include C_2_H_4_, C_2_H_5_OH, CH_3_COOH, and even C_3_H_7_OH.^16^^,^^17^^,^^18^^,^^19^^,^^20^ In recent times, Cu-based CO_2_RR catalysts have made significant progress. Feng et al.^82^ reported an atomic-level Gd-doped Cu_2_O, which achieved an FE of 81.4% for C_2+_ products. The partial current density reached 444.3 mA cm^−2^ under 0.8 V vs. RHE. Chen et al.^83^ accomplished CO_2_RR under acidic conditions by constructing a tandem catalytic system. They combined CoPc with Cu nanoparticles as catalysts and obtained an 82% C_2+_ FE at 800 mA cm^−2^. The one-pass carbon efficiencies reached 90 ± 3% under a 2 mL min^−1^ CO_2_ flow rate. Amidst the remarkable achievements, there are several issues that cannot be overlooked. The C-C coupling process occurring on Cu-based catalysts involves the transfer of multiple electrons and a complex mass transfer process. Electron transfer numbers that can extend from 2e^−^ to 18e^−^, the chaotic reaction path leads to low product selectivity. In 2012, Kendra et al.^84^ found up to 16 types CO_2_RR products on the metallic Cu surface. FE of target products are far from the requirements of industrial production. Moreover, the Cu-based catalyst structure might be *in situ* damaged during the reaction, and its inherent poor stability is its fatal flaw. For example, Jung et al.^85^ have found that Cu_2_O nanoparticles are gradually fragmented during CO_2_RR. Furthermore, the inevitable presence of competing H_2_ evolution reaction (HER) in the cathode can directly lead to a significant reduction in the efficiency of CO_2_RR.^26^^,^^27^^,^^28^^,^^86^ In summary, Cu-based catalysts face various challenges in CO_2_RR. High product selectivity, catalyst stability, electrochemical activity, and Faraday efficiency are all demanding.

The rational design of Cu-based catalysts is crucial to solve the aforementioned problems and achieve industrial production of CO_2_RR. Various Cu-based 2D materials have been developed and successfully applied to CO_2_RR due to their excellent electron and proton transfer function. Based on the mechanism of CO_2_RR over Cu-based catalysts, this section focuses on the applicability of various Cu-based 2D materials in the CO_2_RR and their advantages and shortcomings. The content includes pure Cu NSs, bi/multimetallic 2D Cu-based catalysts, non-metallic composite 2D Cu-based catalysts, porous 2D Cu-based catalysts, and 2D Cu-based single-atom catalysts, to provide a reference for the reasonable design of 2D Cu-based CO_2_RR catalysts.

### Mechanism of CO_2_RR over Cu-based catalysts

**Figure 7:**
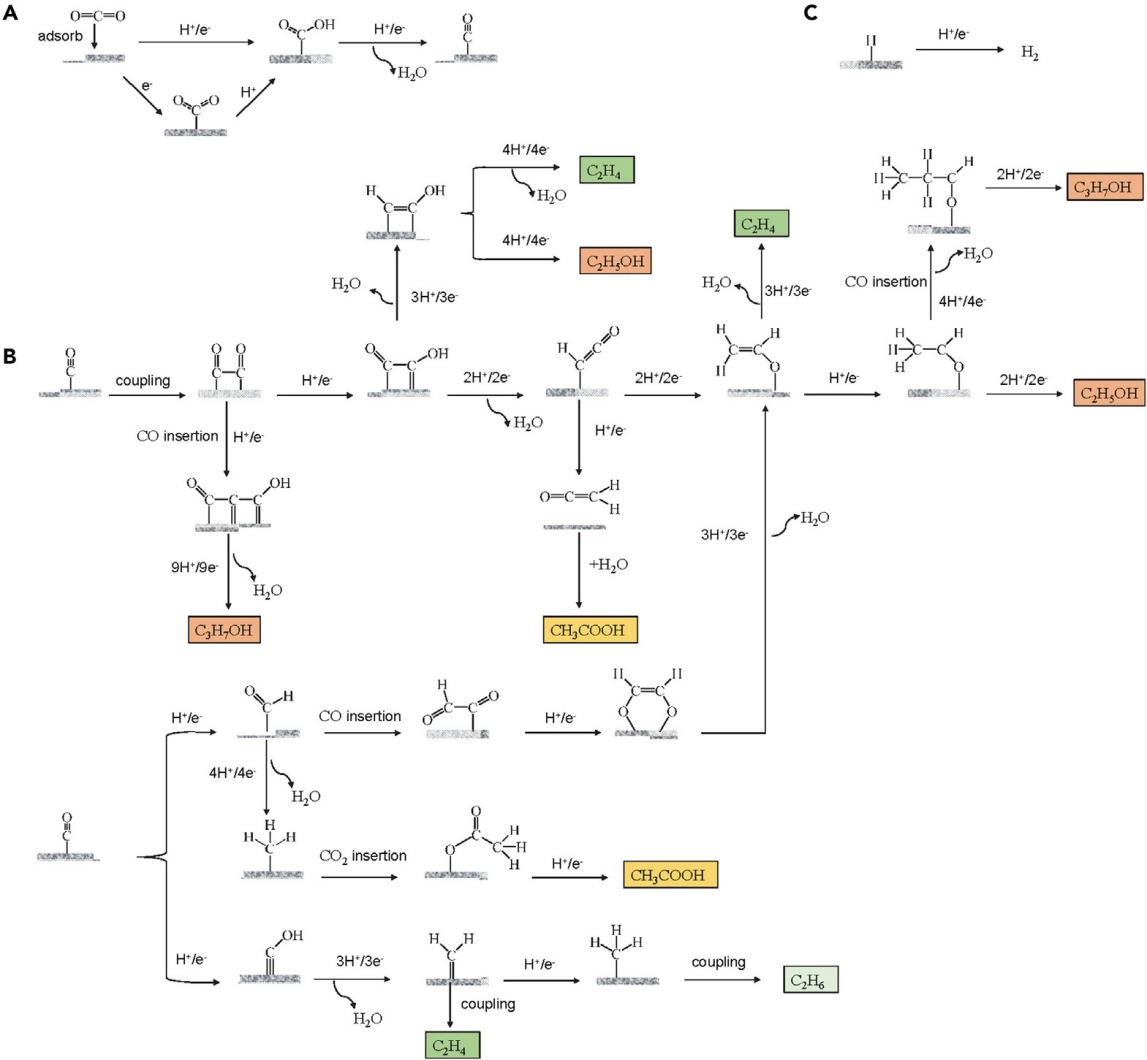
Possible reaction pathways for CO_2_ to C_2+_ products over Cu-based catalysts (A) CO_2_ is reduced to form adsorbed ∗CO via the ∗COOH intermediate. (B) The conversion of ∗CO intermediates determines the final distribution of C_2+_ products. (C) Competing HER in CO_2_RR. (Reproduced with permission from Ref.,^87^ © Chin. J. Catal. 2023).

In addition to generating conventional C_1_ products (e.g., CO, CH_4_, HCOOH), the unique C-C coupling ability of Cu metal is due to mild ∗CO intermediate binding energy. The strong ∗CO adsorption on Ni, Pt, and Fe etc. usually leads to active site poisoning, then the competing HER becomes the major one. ∗CO on Zn, Au, and Ag undergoes direct desorption to gaseous CO products. The negative ∗CO adsorption energy and positive ∗H adsorption energy on Cu are the keys to inhibiting the HER and realizing the C-C coupling process. A comprehensive understanding of the CO_2_RR mechanism over Cu-based catalysts is crucial for achieving highly efficient preparation of C_2+_ products. While the CO_2_RR is not yet fully standardized and can vary under different reaction conditions, examining summarized theories can still be beneficial for understanding the overall reaction process. Figure 7 summarizes the potential pathways involved in CO_2_RR over Cu-based catalysts, which include intricate electron and proton transfer processes. It is widely accepted that CO_2_ first adsorbed as ∗CO_2_ on Cu-based catalysts (Figure 7A) and subsequently forms ∗COOH intermediates in the presence of electrons and H^+^ protons, followed by the conversion to ∗CO by shedding H_2_O.^87^ As shown in Figure 7B, the coupling interactions between the ∗CO are essential for C_2_H_4_, C_2_H_5_OH, CH_3_COOH, C_3_H_7_OH, and other C_2+_ product generation. In addition, ∗CHO generated by the protonation of ∗CO can also participate in the C-C coupling process directly with ∗CO. It can also convert to ∗CH_2_ or ∗CH_3_ and couple with ∗CO_2_ and ∗CHO. There is a relatively small probability that a coupling process will occur between ∗CH_2_ or ∗CH_3_ to get C_2_H_4_ or C_2_H_6_. The step after completion of the C-C coupling process is the key to product selectivity. Taking the intermediate CH_2_CHO∗ obtained after OC∗∗CO or ∗COCHO coupling as an example, hydrogenation can form C_2_H_5_OH or deoxygenation to produce C_2_H_4_. The lower deoxygenation energy barrier makes the FE of C_2_H_4_ on Cu-based catalysts much higher than that of C_2_H_5_OH. Therefore, further modulation of the intermediates obtained by C-C coupling is the key to achieving high product selectivity.^87^^,^^88^

The intermediate C_2_ species can continue to couple with C_1_ species to obtain C_3_ products, such as C_3_H_7_OH. However, the C_2_ intermediates instability state, and more complex reaction mechanism between C_1_ and C_2_ species make it difficult to achieve a high-efficiency process. H^+^ proton transfer runs through the entire CO_2_RR, and HER processes occur in the liquid phase environment to produce H_2_ inevitably (Figure 7C). HER significantly impacts the efficiency of electron utilization and product selectivity, so a rational design of Cu-based catalysts is required to achieve maximum suppression of the HER process. Various types of 2D Cu-based catalysts have been developed to achieve efficient CO_2_RR.^19^^,^^87^^,^^88^

It’s worth noting that even though there have been proposals for generalized mechanisms, different kinds of reduced species may occur on the same Cu-based structure. For instance, when using single-crystal Cu as an electrocatalyst in 0.1 M KHCO_3_ with a fixed current density of 5 mA cm^−2^, CH_4_ was obtained on Cu(111), while Cu(100) generated C_2_H_4_. On Cu(110), CH_3_COO^−^ and C_2_H_5_OH were preferred.^89^ It is crucial to summarize the reaction conditions for specific products when designing Cu-based catalysts for CO_2_RR. Increasing the exposure of Cu(100) to improve C_2_H_4_ selectivity is recommended. For example, Chungseok et al.^90^ have designed step-like Cu nanowires enriched with Cu(100). The nanowires demonstrated a 77.4% ± 3.16% FE of C_2_H_4_ in 0.1 M KHCO_3_ solution. Cu(100) was maintained at 45.40% ± 5.62% during the 200 h stability test. The valence state of Cu also affects C_2_H_4_ selectivity. Mistry et al.^91^ confirmed that Cu^+^ is the active site for its selective ethylene production. Yang et al.^92^ prepared nanoscale porous Cu_2_O spheres to stabilize Cu^+^ and obtained 40% C_2_H_4_ FE under a current density of 355 mA cm^−2^. When the target product is ethanol, modulating the surface adsorption of the carbon-containing intermediates or the hydrogen adsorption energy on the catalyst surface is the crucial to enhance the selectivity. It has been verified that CO coverage favors ethanol production.^93^ Bimetallic catalysts, consisting of Cu and another CO-producing metal (such as Au, Ag, and Zn), were synthesized to enhance ∗CO surface coverage for ethanol production. Yeo et al.^94^ added Zn to a Cu-based catalyst, resulting in the *in situ* generation of CO at the Zn sites. Excess localized CO combined with ∗CHx on the Cu sites can generate C_2_. The Cu_4_Zn structure achieved maximum ethanol selectivity with a 29.1% FE. Moreover, introducing metals such as Pt or Pb can modulate the adsorption energy of H on the catalyst surface, enhancing ethanol selectivity.^95^ Additionally, doping the Cu surface with hydroxides or oxides can alter the H adsorption energy and promote adsorption on the catalyst surface.^96^ When the product expands to C_3_, in the case of n-propanol, an active site for C_1_-C_2_ coupling is essential, since a simple C_1_-C_1_ coupling site can only yield a C_2_ product.^97^ Chen et al.^98^ prepared CuS catalysts with double vacancies, which were able to stabilize the ∗CO and ∗C_2_ intermediates and promote their coupling to produce n-propanol. Moreover, confinement effects also promote the generation of C_3_.^99^^,^^100^ In summary, enhancing the selectivity of target products requires the rational design of catalysts in combination with different reaction conditions. Later, we will discuss the relationship between the structures and effects of various 2D Cu-based catalysts.

### Monometallic Cu nanosheets

**Figure 8:**
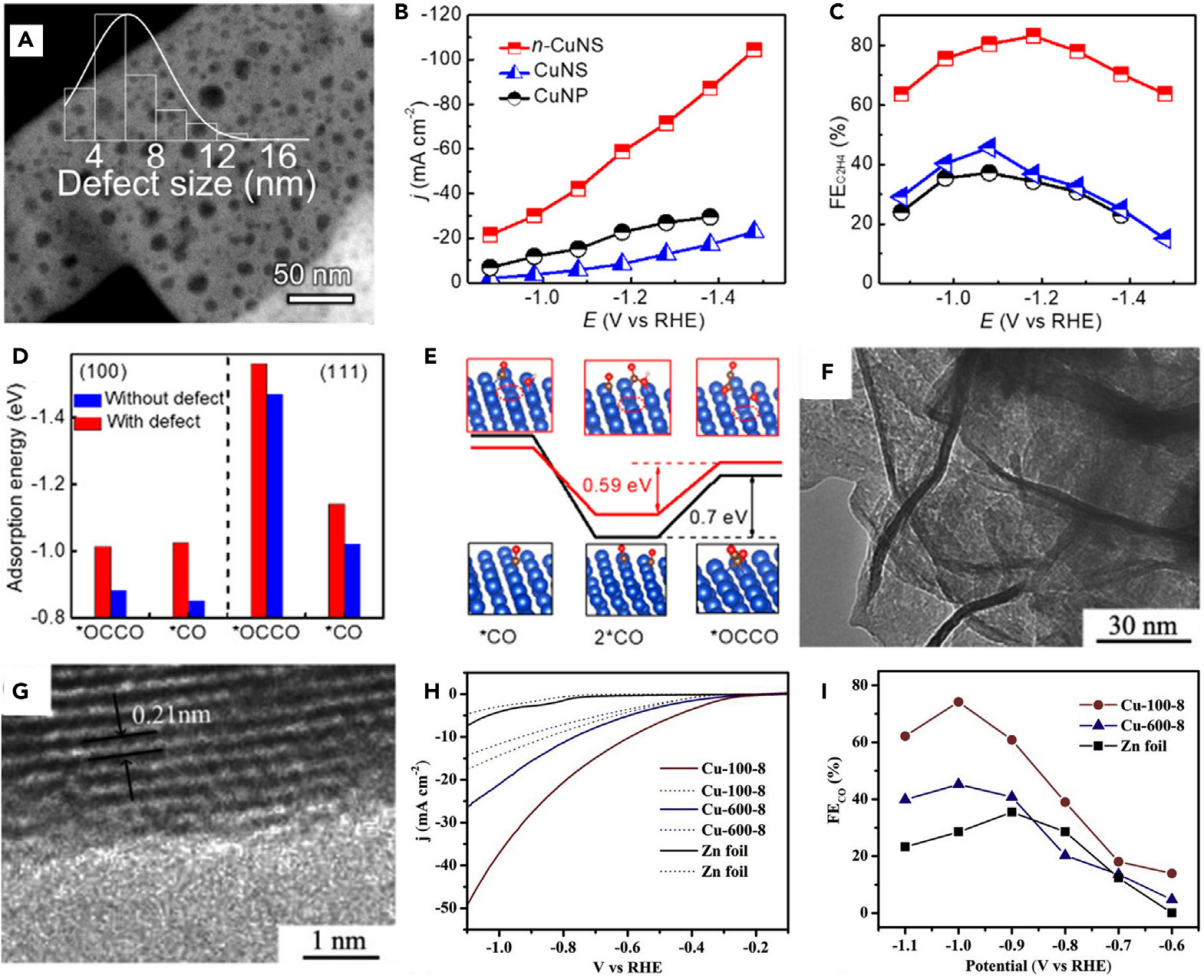
Structure, electrochemical performance and DFT calculations of monometallic Cu nanosheets (A‒E) (A) Size distribution of the nanodefects on n-Cu NSs. (B) Total current density and (C) ethylene FE at various applied potentials for different catalysts. (D) Comparison of adsorption energy of key intermediates that affect selectivity on different facets. (E) Energy diagrams and geometries of CO dimerization on OH^−^ adsorbed and defective Cu(111) of n-Cu NSs (red) and nondefective Cu(111) of Cu NSs (black). Red, gray, white, and blue stand for oxygen, carbon, hydrogen, and copper atoms, respectively.^101^(Reproduced with permission from Ref.,^101^ © J. Am. Chem. Soc. 2020). (F‒I) (F) TEM and (G) HRTEM images of Cu-100-8-P. (H) Polarization curves of the integrated electrodes in the Ar- (dashed line) or CO_2_-saturated (solid line) 0.5 M KHCO_3_ electrolyte. (I) FEs of CO at various applied potentials for the integrated electrodes.^102^(Reproduced with permission from Ref.,^102^ © Appl. Catal. B: Environmental 2019).

The excellent electrical conductivity, large specific surface area, and controlled local surface infiltration of Cu NSs enable them to complete the CO_2_RR. More in-depth work is needed to investigate the selective exposure of crystalline surfaces and the regulation of the structural composition of Cu NSs to achieve a breakthrough in product selectivity. Kang et al.^38^ prepared freestanding triangular 2D Cu NSs with selectively exposed (111) surfaces. The Cu NSs were only 5 nm thick and underwent CO electroreduction in a 2M KOH electrolyte, achieving a 48% FE for acetate under a current density of 131 mA m^−2^. The energy evolution of acetate generation on the Cu(111) crystal plane calculated by DFT demonstrated that the vinyl ketone pathway required for acetate generation was more favorable to occur on the Cu(111) crystal plane. C_2_ products such as ethanol and ethylene were inhibited, thereby enhancing the selectivity of acetate generation. Modulation of intermediates by the structural design of Cu NSs is an ideal way to obtain more C_2_ products, and structural defect engineering is a typical design strategy for heterogeneous catalysts. Zhang et al.^101^ prepared Cu NSs with nano-defects (n-CuNS) with defect sizes ranging from about 2 to 14 nm (Figure 8A) via an electrochemical synthesis process, obtaining 83.2% C_2_H_4_ FE on n-CuNS under 60 mA cm^−2^ current density (Figures 8B and 8C). Electron microscopy, spectroscopy, and DFT theoretical calculations (Figures 8D and 8E) showed that defects on Cu NSs facilitate the adsorption, confinement, and enrichment of reaction intermediates. Defects reduce the reaction energy barrier of ∗CO+∗CO→∗OCCO, and promote the C-C coupling reaction for C_2_H_4_ generation. Xia et al.^102^ achieved the construction of multi-stage pore structures on ultrathin Cu NSs by a simple template method and electrochemical replacement process (Figures 8F and 8G). This porous Cu electrode enabled the enhanced selectivity of CO_2_RR to CO at high current densities. The FE was 74.1% under a 23 mA cm^−2^ current density (Figures 8H and 8I). The instantaneous conversion frequency of the product CO was 0.092 s^−1^. Experiments demonstrated that the hierarchical pore structure significantly promoted the mass transfer and the electron transfer rate of the reaction process, and the formation of vacancies also facilitated the rapid completion of the initial activation process of CO_2_. However, Cu NSs dissolve during CO_2_RR leading to the destruction of pre-designed phases, interfaces, or structures, which limits their realization for breakthroughs in performance and applications. To enhance the stability of Cu NSs in the electroreduction of CO_2_, Liu et al.^103^ proposed a Cu NSs passivation mechanism to achieve a stable electrocatalytic process. An electrolyte containing Cu^2+^ ions enhances the dissolution equilibrium of Cu NSs to achieve the passivation target. It is worth noting that the amount of Cu^2+^ ions must be appropriate, as excessive Cu^2+^ ions will precipitate Cu particles and lead to the damage of Cu NS (111) crystalline surface. Finally, the experimental results demonstrated the passivation process. *In situ* electrochemical quartz crystal microbalance (*in situ* EQCM-D) with dissipation confirmed the stability of the Cu NSs in the presence of Cu^2+^ ions, with only 2.2% of Cu dissolving (this value is 16.6% in the absence of Cu^2+^). The catalytic system completed 10 h of steady operation with 60% CH_4_ selectivity.

### Bimetallic/multimetallic 2D Cu-based catalysts

**Figure 9:**
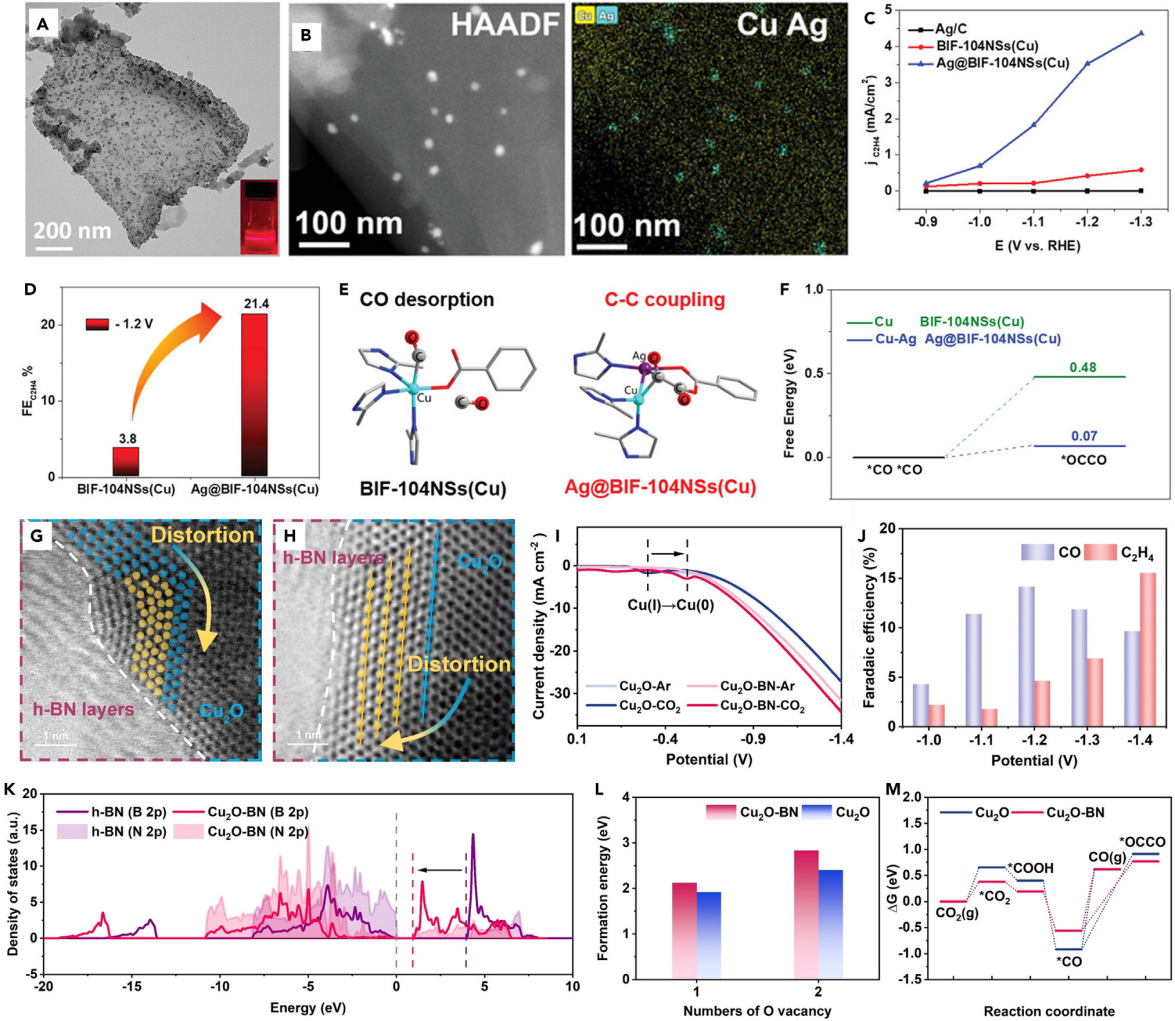
Structure, electrochemical performance and DFT calculations of bimetallic/multimetallic and non-metallic composite 2D Cu-based catalysts (A‒F) (A) TEM image of Ag@BIF-104 NSs(Cu) electrocatalysts (the inset displays the Tyndall effect of a colloidal suspension of Ag@BIF-104 NSs(Cu)). (B) HAADF STEM image and corresponding mapping of Cu Ag overlay. (C) Partial current density toward C_2_H_4_ products over Ag@BIF-104 NSs(Cu), BIF-104NSs(Cu), and Ag/C electrocatalysts in CO_2_ saturated electrolyte. (D) FE of C_2_H_4_ in CO_2_RR on Ag@BIF-104 NSs(Cu) and BIF-104NSs(Cu) at −1.2 V vs. RHE. (E) Calculated structures of CO desorption for Cu sites on BIF-104 NSs(Cu), and the C-C coupling for Cu-Ag sites on Ag@BIF-104 NSs(Cu). (F) Free energy diagram of ∗OCCO intermediates for CO_2_RR on Cu sites and Cu-Ag sites of Ag@BIF-104 NSs(Cu).^80^(Reproduced with permission from Ref.,^80^ © Adv. Energy Mater. 2023). (G‒M) (G) and (H) The disarrangement and dislocation of Cu atoms in the vicinity of the interface. (I) LSV curves of Cu_2_O and Cu_2_O-BN in Ar and CO_2_ saturated 0.5 M KHCO_3_ solution. (J) CO and C_2_H_4_ FE of Cu_2_O-BN at different applied potentials during 1 h of electrolysis in 0.5 M KHCO_3_ electrolyte. (K) B 2p and N 2p bands of Cu_2_O-BN and h-BN. (L) Computed oxygen vacancy formation energy in Cu_2_O(111) and Cu_2_O-BN models. (M) Free energy diagram for CO_2_RR to different intermediates on Cu_2_O and Cu_2_O-BN.^107^(Reproduced with permission from Ref.,^107^ © Angew. Chem. Int. Ed. 2022).

Besides modulating the crystalline surface or surface defects of Cu NSs to get higher CO_2_ conversion and product selectivity, introducing foreign metal elements can make 2D Cu-based catalysts versatile. For example, Cu-based alloy catalysts can better accomplish reactant adsorption and activation, intermediate species generation, and target product desorption. The synergistic effect between different metal elements can further accelerate proton and electron transfer, regulate the energy barrier of reaction evolution, and achieve a high-efficiency CO_2_RR. As previously mentioned, Chen et al.^39^ used large-area 2D Cu_2_Te NSs prepared by CVD to realize the CO_2_RR in a flowing electrolytic cell with 63% methane FE under 300 mA cm^−2^ current density. Experimental and theoretical calculations demonstrate that relative to the pure Cu catalyst, the introduction of Te makes Cu_2_Te NSs more capable of transforming CO_2_ to CH_4_. In 2017, Zheng et al.^104^ reported the synthesis of hybrid Cu/Ni(OH)_2_ NSs with atomic-scale thickness by a solvothermal process. The catalyst achieved a CO_2_RR at a low overpotential (0.39 V). FE of the product CO reached 90% with a 4.3 mA cm^−2^ current density. Formate salt added during the synthetic process can stabilize Cu in the air for several months, and the Cu/Ni(OH)_2_ NSs also completed a continuous run of 22 h without any decay in activity. Pd is the only catalyst able to complete the electroreduction of CO_2_ to formate at near-zero overpotential. But the poisoning of by-product CO limits its application, and alloying it with Cu is an ideal route. Li et al.^105^ prepared a 2D bimetallic PdCu nanodendrite catalyst (nd-PdCu) by a solvothermal process using octadecyltrimethylammonium chloride (OTAC) as a surfactant. This 2D fractal nanodendrite structure provided an electrochemical specific surface area of 51.0 m^2^ g^−1^ and abundant catalytic active sites. The conversion of CO_2_ to formate at a cathodic potential of −0.4 V was stable. Theoretical calculations demonstrate that PdCu alloying weakens the surface adsorption of ∗CO and solves the catalyst poisoning problem, meanwhile promoting the adsorption of the intermediate species ∗OCHO to favor the formate generation. Similar to the alloying feature, the doping between heterogeneous metal elements is also beneficial for the roles of intermediate species modulation and promoting reactant adsorption. Zhang et al.^106^ solved the problem of low reaction current of Bi-based materials by constructing 2D Bi NSs doped with Cu. The FE of CO_2_RR to formic acid under alkaline conditions peaked at 96.1%. The current density was 1132 mA cm^−2^ under −0.86 V and remained stable for 100 h. The introduction of Cu decreased the adsorption energy barrier of CO_2_ (from 0.433 eV to 0.428 eV) and inhibited the HER process in addition to achieving a stable Bi NSs structure. In addition to the process of alloying mentioned above, designing the spatial configuration of heterogeneous metals can realize tandem reactions. As shown in Figures 9A and 9B, Shao et al.^80^ achieved an efficient tandem reaction process by constructing catalysts with Cu-Ag bimetallic sites (Ag@BIF-104 NSs(Cu)) on ultrathin boron imidazole ester backbone (BIF) NSs. Specifically, by introducing Ag nanoparticles attached to the highly ordered benzoate ligand-modified Cu sites, the Ag sites can successfully adsorb CO_2_ to ∗CO for subsequent transfer to the Cu sites (Figures 9E and 9F). Eventually, the coupling of ∗CO locally enriched in Cu-Ag sites resulted in ethylene. Ag@BIF-104 NSs(Cu) achieved 21.43% ethylene FE. Compared with the single Cu site catalyst (BIF-104 NSs(Cu)) of only 3.82%, the FE of ethylene was significantly improved (Figures 9C and 9D). The heterometallic introduction significantly enhances the activity and selectivity of the CO_2_RR. But the shortcoming lies in the tendency of C_1_ product generation. It is the consequence of the modulation of the reaction intermediate species by the heterometal or the absence of Cu as the active site for the C-C coupling process. Therefore, it is worthwhile to enhance the selectivity of Cu-based catalysts for C_2+_ products by introducing a non-metallic element.

### Non-metallic composite 2D Cu-based catalysts

Oxygen is the most typical nonmetallic element introduced into Cu-based catalysts to form CuO or Cu_2_O,^20^ which enables the modulation of the electronic structure of Cu by regulating its oxidation state and ultimately improves the catalytic performance. Moreover, the presence of oxygen elements can control the oxidation state of the metal center after *in situ* reduction.^26^ In 2022, Wu et al.^40^ reported the preparation of CuO NSs for CO_2_RR to ethylene process by electrochemical deposition strategy. The CuO NSs reorganized during the CO_2_RR process, forming high-density grain boundaries (GBs). Cu atoms located in the GBs region with low coordination form a Cu^+^/Cu^0^ interface and serve as the active site for the reduction of CO_2_ to ethylene at low potential (−0.52 V) with a local current density reached an industrial grade of 173 mA cm^−2^. The FE of ethylene is 62.5%, corresponding to a record-high half-cell cathode energy efficiency of 41%. *in situ* Raman spectroscopy and DFT calculations reveal the role of the Cu^+^/Cu^0^ interface. The interface improved the coverage of ∗CO and reduced the energy barrier for the dimerization of ∗CO to ethylene. Adjusting the electronic structure of Cu can also be achieved by introducing interactions between the nonmetallic components and the oxidized Cu. Numerous experimental results demonstrate that a stable single oxidized Cu^+^ is essential for the CO_2_RR and the selectivity of the C_2+_ product. Hexagonal boron nitride (h-BN) NSs have excellent electron transport ability and structural stability. The 2D hexagonal boron nitride (h-BN) NSs composite of Cu_2_O catalyst (Cu_2_O-BN) prepared by Yan et al.^107^ successfully achieved the stabilization of Cu^+^ species in the catalyst by exploiting the electronic interactions between the components (Figures 9G and 9H). The electrons accumulated at the Cu_2_O site during the reaction can be transferred to the electron-friendly h-BN to avoid direct electron attack on the Cu-O bond to maintain high activity. Such a result comes from the electronic interaction between the Cu 3days and the B 2p electron orbital. DFT calculations demonstrate this interaction not only enhances the Cu-O bond strength but also has lower activation energy for the adsorption of CO_2_ and the dimerization energy of ∗CO (Figures 9K‒9M). As shown in Figures 9I and 9J, the Cu_2_O-BN catalyst has a 1.62 times higher C_2_H_4_/CO ratio at a negative potential of −1.4 V compared to Cu_2_O and can maintain a stable operation for 14 h. Besides directly participating in the CO_2_RR, the non-metallic component can also modulate the surface and Cu site spatial structure to facilitate the reaction. Wu’s group^108^ successfully synthesized ultrathin 2D Cu_2_-xSe catalysts (VSe-Cu_2_-xSe) containing abundant selenium vacancies by solvothermal. The lattice stress due to the presence of Se vacancies induces a significant shortening of the nearby Cu-Cu spacing (from 4.16 Å to 2.51 Å), and this change effectively adjusts the local charge distribution. The decrease in the valence state of Cu atoms and the enhanced electron-giving ability trigger reduce the Gibbs free energy of the asymmetric ∗CO-∗CHO coupling process and enhance the reactivity. The ethanol FE of VSe-Cu_2_-xSe is higher than that of Cu_2_-xSe catalysts in the voltage range of −0.4 to −1.6 V, and its ethanol FE at −0.8 V is highest at 68.1%. The stability test for 6 h showed less than a 5% reduction in FE and no significant structural changes. Xie et al.^109^ also reported a swelling-resistant anion exchange ionomer (AEI) composite Cu NSs catalyst (AEI-OD-Cu NSs) for CO_2_RR to C_2+_ at industrial-grade current densities through local environment optimization. The most remarkable feature of AEI is the regulation of local pH and water content. AEI has an anti-swelling backbone to inhibit competitive H_2_ evolution by controlling the water content, thus facilitating the proton-electron transfer step of C_2+_ production. *In situ* Fourier transform infrared spectroscopy and theoretical calculations showed that a higher local pH formed by OH^−^ accumulated in the -N(CH_3_)^3+^ group in AEI. Higher local pH can reduce the energy barrier of the crucial step (COCO∗ to COCOH∗) from 0.08 eV to 0.04 eV, thus promoting the production of C_2+_ products. Ultimately, the AEI-OD-Cu NSs catalyst achieved 85.1% C_2+_ FE at 800 mA cm^−2^ current density and a half-cell power conversion efficiency of more than 50%, which is an excellent reference for realizing the industrial application of this reaction.

### Porous 2D Cu-based catalysts

**Figure 10:**
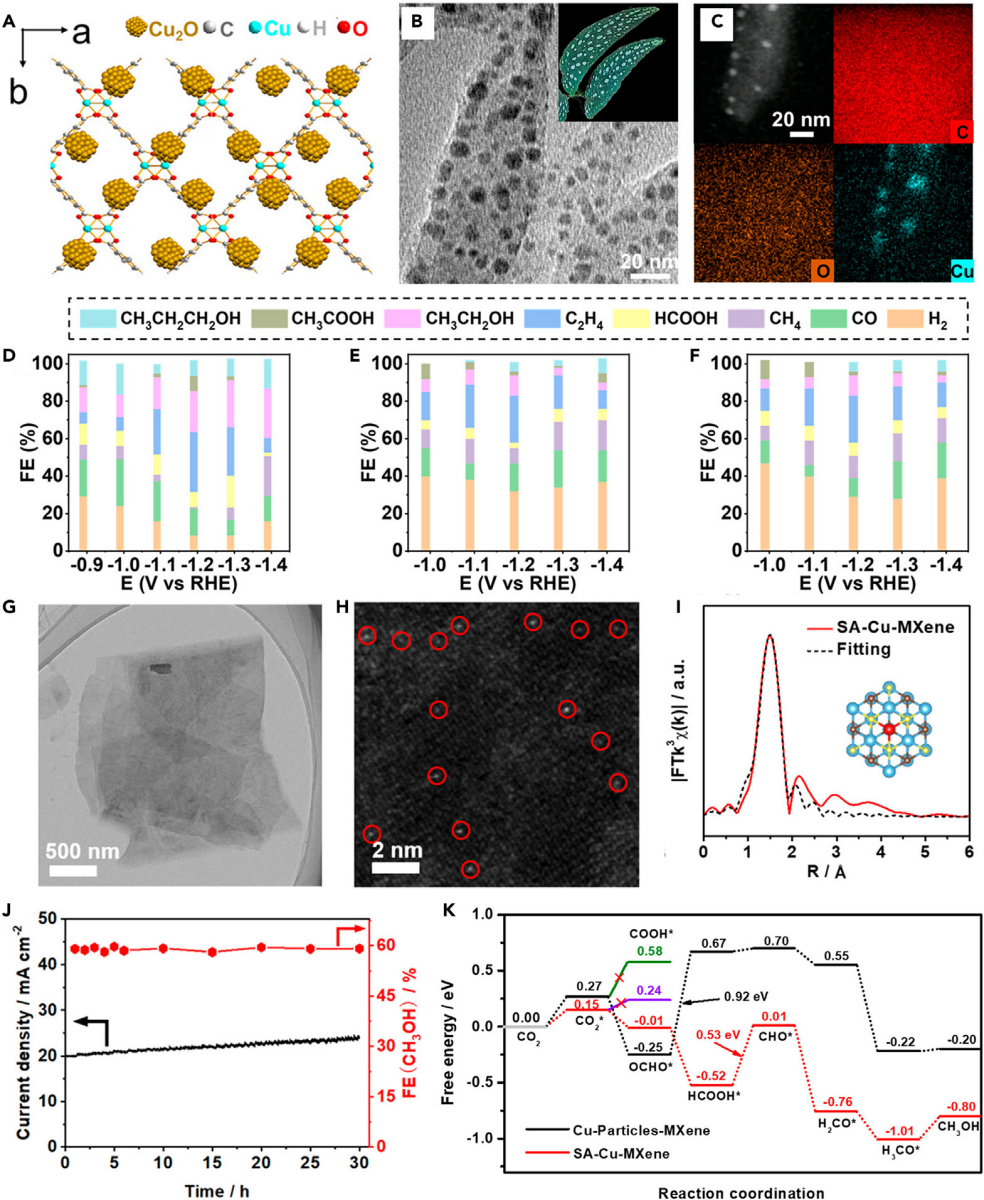
Structure, electrochemical performance and DFT calculations of porous and single-atom 2D Cu-based catalysts (A‒F) (A) Structural schematic diagram of Cu_2_O@Cu-BDC. (B) TEM image of Cu_2_O@Cu-BDC. The inset in (B) is the STEM image of the NSs. (C) HAADF-STEM image and EDS mapping of Cu_2_O@Cu-BDC. FE of different products for (D) Cu_2_O@Cu-BDC, (E) Cu-BDC, and (F) Cu_2_O electrodes as a function of applied potential.^113^(Reproduced with permission from Ref.,^113^ © Nano Lett. 2023). (G‒K) (G) TEM image of SA-Cu-MXene. (H) AC HAADF-STEM image of SA-Cu-MXene. (I) EXAFS curves between the experimental data and the fit of SA-Cu-MXene (inset is the fitted structure). (J) Current-time responses and corresponding FE (CH_3_OH) of SA-Cu-MXene for CO_2_RR at −1.4 V for 30 h. (K) Free energy diagram of CO2 to CH3OH on Cu-O3 structure.123(Reproduced with permission from Ref.,^116^ © ACS Nano 2021).

Porous materials have been widely developed and applied to electrocatalytic processes with remarkable effects due to their large specific surface area and numerous arranged pore structures. Porous structures can provide abundant active sites and localized reaction spaces for reactions and regulating the products. Porous 2D Cu-based catalysts combining porous properties with 2D characteristics have shown a new direction for enhancing CO_2_RR performance.^110^^,^^111^^,^^112^ Metal-organic frameworks (MOFs), as a typical class of porous materials, become a popular non-homogeneous catalyst in recent years. 2D MOFs exhibit low mass transfer diffusion resistance and easily accessible active sites that can effectively promote the CO_2_RR, and the stable coordination structure can ensure that the activity does not decay rapidly. In addition, the controlled pore structure of 2D MOFs can produce a domain-limiting effect to promote the C-C coupling process and increase product selectivity. As shown in Figures 10A–10C, Liu et al.^113^ generated a Cu_2_O nanoparticle-modified 2D Cu-BDC (Cu_2_O@Cu-BDC) by a simple wet chemical process. Cu_2_O@Cu-BDC constructed a rich Cu_2_O/Cu-BDC heterogeneous interface. This interface achieved the C-C coupling process by stabilization of Cu^+^ and optimized the adsorption of key intermediate species (∗CHO, ∗COH, ∗CO). High C_2+_ FE of 72.1% and 58.2% were obtained in the H-type and flow-through electrolytic cells, respectively (Figures 10D–10F). Another outstanding advantage of 2D Cu-based MOF electrocatalysts is their ability to design multi-metal sites to enhance reaction activity and product modulation. For instance, Feng et al.^114^ prepared a 2D c-MOF electrocatalyst (PcCu-O_8_-Zn) with Cu-Zn bimetallic sites by solvent heat. It exhibited high selectivity (88%) for CO_2_-CO conversion and a high turnover frequency (TOF) of 0.39 s^−1^ at −0.7 V with excellent stability. The ZnO_4_ and CuN_4_ site in the PcCu-O_8_-Zn structure has the functions of activating CO_2_ and promoting protonation, respectively. ZnO_4_ and CuN_4_ site synergy is the key to proving the performance. Beyond the efficient CO_2_RR through this stationary structure, 2D Cu-based MOFs can also regulate the activity and selectivity through the dynamic evolution of the *in situ* process. Representatively, Han et al.^115^ prepared a 2D Cu-MOF film (HKUST-1) by an electrodeposition process. During the CO_2_RR process, HKUST-1 undergoes structural reconfiguration with increasing electrolysis time. After 15 min of electrolysis, the structure of HKUST-1 transforms into a spherical shape composed of numerous nanofragments with a very high Cu^+^/Cu ratio, promoting CO_2_ activation and completing C-C coupling to obtain CH_3_CH_2_OH and C_2_H_4_. The current density reaches 19.2 mA cm^−2^ at −0.98 V, and the FE of the C_2_ product is 58.6%.

As an emerging porous material, covalent-organic frameworks (COFs) consist of some light elements (H, B, C, N, O, etc.) and form covalent bonds, then assemble them into highly crystalline and long-range ordered mesh structures.^112^ COFs have better stability than MOFs and can achieve excellent electrical conductivity by planar π-conjugation.^117^ Therefore, 2D Cu-based COFs are potential candidates for achieving efficient CO_2_RR. Lan et al.^41^ used a functional material stripping reagent Dct (2,4-diamino-6-chloro-1,3,5-triazine) to strip bulk COF to obtain large-area ultra-thin Cu-based COF NSs (Cu-Tph-COF-Dct). DFT calculations demonstrated the combination of Dct groups in the exfoliation reagent with amino and triazine groups effectively enhanced the CO_2_ adsorption and activation of Cu-Tph-COF-Dct. It also stabilized the reaction intermediate and increased the concentration of CO near the Cu catalytic active site. Cu-Tph-COF-Dct achieved excellent FE CH_4_ (∼80%) at −0.9 V with a current density of 220.0 mA cm^−2^. The C_2_ product is equally capable of being generated on a 2D Cu-based COF. Liao et al.^118^ demonstrated a stable and conductive 2D phthalocyanine-based COF (PcCuTFPN) as an electrocatalyst for CO_2_ reduction to acetate. The single product FE was 90.3% at −0.8 V with a current density of 12.5 mA cm^−2^. The catalyst was operated for 80 h continuously without significant degradation. Theoretical calculations and *in situ* IR spectroscopic analysis indicated that the isolated Cu phthalocyanine active site with high electron density facilitated C-C coupling of ∗CH_3_ and highly selective acetates formation with CO_2_.

Besides MOFs and COFs, 2D Cu-based catalysts based on other porous structures have also shown good CO_2_RR ability. For example, Yeo et al.^119^ used porous 2D MgAl LDH as a substrate to complete the dispersion of CuO and further reduced it to prepare 2D Cu-MgAl LDH catalysts. The accelerated reaction rate was derived from the enhanced gas transfer diffusion by the porous structure of LDH NSs. Current densities of C_2+_ products reached −1251 mA cm^−2^ at an overpotential of −0.7 V, and the partial current densities of C_2+_ organic alcohols and ethylene reached 369 mA cm^−2^ and 816 mA cm^−2^, respectively. 2D carbon-based porous materials have excellent electrical conductivity and unique electronic properties, among which nitrogen-doped porous carbon shows excellent electrocatalytic functions.^120^^,^^121^ Cu-based 2D porous C/N complex materials have good performance in CO_2_RR. For example, graphene modified with Cu can be used for the CO_2_RR. Zang et al.^122^ prepared a Cu-N-doped graphene catalyst by CVD process to finish the electroreduction of CO_2_ to ethanol at −0.8 V. The ethanol selectivity is significantly enhanced compared to pure Cu and simple graphene-loaded Cu catalysts because the interface formed by Cu and N-doped graphene facilitates the dimerization process of ∗CO.

### 2D Cu-based single-atom catalysts

The construction of atomic-scale catalysts is a research hotspot in the catalytic field. Various catalytic scenarios used single-atom catalysts due to their unique geometric, electronic structures, quantum size effect exhibited at the atomic scale, surface effect, and metal-support interactions.^123^^,^^124^ Researchers have reported 2D Cu-based single-atom catalysts with independent and tunable active sites for the CO_2_RR. As mentioned in the previous section, Cu in 2D MOF or 2D COF structures sometimes engages in the CO_2_RR under the single-atom state,^114^^,^^118^ so we will not repeat the discussion of these types. This section will mainly discuss some other types of 2D Cu-based single-atom catalysts. For example, Yang et al.^116^ utilized a typical 2D material, Mxene, as a substrate to prepare Cu-based single-atom catalysts (SA-Cu-MXene). The synthesis process used the quaternary MAX phase (Ti_3_(Al_1-x_Cu_x_)C_2_) as the origin species and successfully obtained SA-Cu-Mxene after selective etching and ultrasonic stripping processes. Cu atoms during etching were immobilized as Cu-O bonds on the surface of the Ti layer containing Cl functional groups to achieve atomic dispersion (Figures 10G–10I). X-ray absorption spectroscopy and DFT calculations demonstrated that this coordination-unsaturated Cu^δ+^ species (0 < δ < 2) can lower the energy barrier of the decisive step (HCOOH∗ → CHO∗) (Figure 10K). SA-Cu-Mxene completed the CO_2_RR to methanol with a current density of 21.3 mA cm^−2^ at −1.4 V, and the FE of methanol can stabilize above 58% within 30 h (Figure 10J). The representative 2D structure of carbon-based 2D materials, graphene/graphene, was also developed for Cu-based single-atom catalysts. Wang et al.^42^ constructed Cu-C bonds by *in situ* adsorption reduction process to anchor Cu single-atom sites on graphene, and the stable coordination structure avoided the agglomerate and erosion of Cu sites. The +1 valence Cu can also promote the formation of intermediate ∗OCHO and inhibit the hydrogen precipitation reaction and formation of CO. The catalyst achieves 81% FE electrocatalytic CO_2_ to CH_4_ in a flow cell electroreduction system, with a local current density of 243 mA cm^−2^ 2D metal-N-C (M-N-C) single-atom structures derive from carbon- or nitrogen-containing precursors (urea, metal imidazole salt MOF, etc.).^125^^,^^126^ This classical single-atom structure allows the flexible design of metal atom centers to form Cu-N-C structures. Cometto et al.^127^ designed Cu-based single-atom catalysts using 2D g-C_3_N_4_ as support and completed the CO_2_RR in a KHCO_3_ electrolyte to obtain formates. Rose et al.^128^ accomplished attractive work using 2D Cu-N-C catalysts by completing the evolution of the Cu-N_4_ structure to Cu-N_4_-xC_x_ at 1000°C. DFT calculations explained the dual activity of Cu-N_4_-xCx for the electroreduction of CO_2_ and NO_3_^−^ and finally realized the electrocatalytic route from CO_2_ to urea. Under a current density of 27 mA cm^−2^ at 0.9 V, the FE of urea reached 28% with a yield of 4.3 nmol s^−1^ cm^−2^. Some unconventional 2D structures (e.g., single-atom layer materials formed by cross-linking polymerization of porphyrin rings as structural matrix) are suitable for precisely tuning the coordination metal atoms to achieve complete homogeneity and high order of the single metal sites. Wang’s group^129^ has synthesized porphyrin-based monoatomic layer materials with a thickness of only 2.8 Å by hydrothermal method. The carboxyl functional group on the metalloporphyrin monomer coordinates with Cu^2+^ and then gets cross-linked to form the monoatomic layer assemblies. The prepared porphyrin-based monoatomic layers with Cu-N_4_ as the catalytic site exhibit high HCOO^−^ and CH_4_ selectivity during CO_2_RR (FE of 80.9% and 11.5% at −0.7 V, respectively).

Performance of different types of 2D Cu-based catalysts are summarized in Table 2.

**Table 2:** Summary of the performance of different types of 2D Cu-based catalysts

Catalyst classification	Representative catalysts	Product	Current density/mA cm^−2^	FE/%	Reference
Monometallic Cu nanosheets	2D Cu NSs (111)	acetate	131	48	Kang et al.^38^
	n-CuNSs	ethylene	60	83.2	Zhang et al.^101^
Bimetallic/multimetallic Cu-based catalysts	Cu_2_Te NSs	methane	300	63	Chen et al.^39^
	Ag@BIF-104 NSs(Cu)	ethylene	∼16	21.43	Shao et al.^80^
Non-metallic composite Cu-based catalysts	CuO NSs	ethylene	173	62.5	Wu et al.^40^
	VSe-Cu_2_-xSe	ethanol	10.96	68.1	Wu et al.^108^
Porous Cu-based catalysts	HKUST-1	ethanol; ethylene	19.2	58.6	Han et al.^115^
	PcCuTFPN	acetate	12.5	90.3	Liao et al.^118^
Cu-based single-atom catalysts	SA-Cu-Mxene	methanol	21.3	58	Yang et al.^116^
	Cu-N_4_-xCx	urea	27	28	Rose et al.^128^

### Conclusion and outlook

Up to now, the utilization of 2D Cu-based catalysts in CO_2_RR has attracted widespread attention. The attributes of rapid electron conduction, low mass transfer resistance, abundant active sites, and unique C-C coupling make 2D Cu-based catalysts outstanding and promising for various applications. This review presented different synthetic strategies, “top-down” and “bottom-up” routes are suitable for specific preparation scenarios. We also discussed the performance of several types of 2D Cu-based catalysts in CO_2_RR, which has important implications for catalyst structure design and product modulation. However, although the 2D Cu-based catalysts have achieved significant improvements in catalytic performance, their industrial applicability remains distant. The prospects for industrial-scale CO_2_RR using 2D Cu-based catalysts still have many opportunities and challenges, mainly including the following aspects.

(1) From the perspective of synthesis routes, both the “top-down” route (e.g., exfoliation synthesis process) and the “bottom-up” route (e.g., CVD) have the shortcomings of low yield in the synthesis process, making the 2D Cu-based catalysts difficult to achieve industrial-scale preparation. Although researchers have developed strategies such as solvothermal processes to improve yields, the additional energy and material consumption makes the whole process uneconomical. These classical methods remain deficient in the controllable synthesis of 2D structures. The lack of precursors also limits the scale-up preparation of 2D Cu-based catalysts. In the future, ionic or molecular intercalation technology, low-temperature growth procedures, artificial intelligence-based material design, and fast and reliable nondestructive characterization are the essential tools for the industrial preparation of Cu-based 2D materials.
(2) The efficiency of CO_2_RR is a prerequisite for its commercial application. A current density over 200 mA cm^−2^ is a baseline, but most catalysts below 100 mA cm^−2^ in H-type electrolytic cells. Flow and membrane electrode cells promote current density effectively, but high costs and the susceptibility of gas diffusion electrodes to deactivation limit their utilization. Therefore, researchers might formulate new electrolytic devices and gas diffusion electrodes to raise the low current density challenges. In addition, although the 2D Cu-based catalysts can accomplish high selectivity of C_1_ and C_2_ clusters, the FE for a specific C_2+_ product is still below 80%. A combination of experimental test techniques and theoretical calculations is needed to guide the regulation of reaction intermediates and optimization of reaction paths to achieve high product selectivity. We should not neglect the operational lifetime either, as most Cu-based catalysts have a sustained stability time of fewer than 100 h. Liquid products, electrolyte type, and environmental pH can cause Cu-based catalysts structural damage. Advanced *in situ* observation techniques and modifications are needed to explore the deactivation mechanism and enhance its stability. Exploitation of electronic interactions is a potential solution to achieve high stability. Valery et al.^130^ identified an *in situ* dissolution-reprecipitation process of Cu nanoparticles during CO_2_RR. The introduction of Ga, which has a higher oxophilicity and lower electronegativity, was the solution to avoid Cu oxidation. Ligand modification is a technique that can maintain the high stability of Cu-based catalysts. Gao et al.^131^ utilized polydopamine (PDA) coordinated with Cu^2+^. During the CO_2_RR, the PDA ligands can self-assemble onto the surface of Cu nanoparticles, which prevents further remodeling, ultimately maintaining high dispersion and stability.
(3) A reliable reaction mechanism is the basis for effectively regulating the reaction and can guide the industrial-scale CO_2_RR. However, the mechanism of CO_2_RR is still unclear and controversial, and most existing theories are summarized based on numerous experimental results and DFT calculations. Advanced characterization techniques are needed to demonstrate the real active sites and reliable reaction pathways. Furthermore, machine learning provides an artificial intelligence route to develop high-performance electrocatalysts.^132^^,^^133^^,^^134^ DFT calculations and advanced *in situ* experiments combined with machine learning can automatically, efficiently, and accurately investigate the structural and physicochemical properties. It can also predict and optimize the design of new catalysts in terms of their composition, structure, active sites, and reaction pathways, providing a faster, more accurate, and feasible approach for exploring catalytic materials. Based on suitable models, ideal algorithms, and modeling methods, machine learning can accomplish the development of novel CO_2_RR catalysts.

In summary, 2D Cu-based catalysts have tentatively exhibited excellent catalytic performance in CO_2_RR. While there are remaining many pitfalls and challenges, the ongoing rigorous research is poised to pave the way for the eventual commercialization of this technology. It is essential to produce 2D Cu-based catalysts on a large scale for direct utilization of CO_2_. In addition, CO_2_RR is a systematic process for simultaneously developing the electrolytic cell, ion exchange membrane, and gas diffusion electrode (GDE). The next generation of electrolytic cells and ion exchange membranes must meet the performance indicators for commercialization and expandability. The GDE should have a breakthrough in mass transfer efficiency and intermediate coverage regulation. Additionally, incorporating auxiliary facilities with artificial intelligence is a technical reserve to ensure the industrialization of the entire CO_2_RR system. We firmly believe that in the future, 2D Cu-based catalysts can hold immense significance for energy sustainability and low-carbon manufacturing.
